# Probability genotype imputation method and integrated weighted lasso for QTL identification

**DOI:** 10.1186/1471-2156-14-125

**Published:** 2013-12-30

**Authors:** Nino Demetrashvili, Edwin R Van den Heuvel, Ernst C Wit

**Affiliations:** 1 Johann Bernoulli Institute for Mathematics and Computer Science, University of Groningen, Groningen 9747 AG, The Netherlands; 2 Department of Epidemiology, University Medical Center Groningen, University of Groningen, Groningen 9700 RB, The Netherlands

**Keywords:** Arabidopsis, Germination traits, QTL mapping, Recombinant inbred line (RIL), Binary genotypes, Likelihood-based genotype imputation, Sparse variable selection, Weighted lasso

## Abstract

**Background:**

Many QTL studies have two common features: (1) often there is missing marker information, (2) among many markers involved in the biological process only a few are causal. In statistics, the second issue falls under the headings “sparsity” and “causal inference”. The goal of this work is to develop a two-step statistical methodology for QTL mapping for markers with binary genotypes. The first step introduces a novel imputation method for missing genotypes. Outcomes of the proposed imputation method are probabilities which serve as weights to the second step, namely in weighted lasso. The sparse phenotype inference is employed to select a set of predictive markers for the trait of interest.

**Results:**

Simulation studies validate the proposed methodology under a wide range of realistic settings. Furthermore, the methodology outperforms alternative imputation and variable selection methods in such studies. The methodology was applied to an Arabidopsis experiment, containing 69 markers for 165 recombinant inbred lines of a F8 generation. The results confirm previously identified regions, however several new markers are also found. On the basis of the inferred ROC behavior these markers show good potential for being real, especially for the germination trait G_max_.

**Conclusions:**

Our imputation method shows higher accuracy in terms of sensitivity and specificity compared to alternative imputation method. Also, the proposed weighted lasso outperforms commonly practiced multiple regression as well as the traditional lasso and adaptive lasso with three weighting schemes. This means that under realistic missing data settings this methodology can be used for QTL identification.

## Background

Quantitative traits are traits that vary continuously. The phenotype of a quantitative trait (QT) is the cumulative result of several genes, their interactions and the environment. Regions within genomes that contain genes associated with a particular QT are known as quantitative trait loci (QTL) [1]. The major biological question is to identify the QTL associated with variation in traits. Understanding the genetic architecture of quantitative traits is important for animal and plant breeding, medicine, and evolution. For example, plant breeders can use the QTL identified for seed quality to select and breed plants with certain desirable characteristics. Molecular markers are specific fragments of DNA that represent the genetic differences between individual organisms or species [1]. Development of molecular (or genetic) markers creates new opportunities for QTL identification. Markers are not usually targets themselves but act as “flags” for genes controlling the trait. Molecular markers that are closely located and tightly linked to genes that control the trait are referred to as “tags”.

The process of coupling the phenotype (i.e. trait measurements) and genotype (i.e. molecular markers) data followed by QTL analysis is known as QTL mapping. The aim of QTL mapping is to identify the markers which are tightly linked to genes affecting the trait as well as to estimate the magnitude of their effects. Most methods consider repeated single QTL models, but it is now understood that modeling multiple QTLs simultaneously, as we consider in this paper, is superior to single QTL models [2]. Often both the phenotype and genotype data are incomplete. Though imputation methods for phenotype data in the context of QTL mapping is quite well-developed [3,4], there is less consensus on imputation of missing genotype data, due to its categorical nature. Two major strategies for genotype imputation are based on: (1) a maximum likelihood and (2) multiple imputation strategies [5]. Although multiple imputation is potentially flexible, it tends to be slow for large fraction of missing values. Therefore, we propose a likelihood-based method. In the context of QTL mapping, existing genotype imputation methods use phenotype data and multiple generation information to obtain a conditional probability of a missing genotype [6]. These methods are design-specific and lack generalizability [6,7]. Most commonly, the missing genotypes are replaced with predicted values that are based on the observed genotypes at neighboring markers, as in the multiple QTL mapping (MQM) algorithm [8,9].

Due to the roughly Markov structure of the meiosis process, we introduce a probability imputation method for markers with binary genotypes that includes information only from immediate neighbors. This method is applied to recombinant inbred line (RIL) experiment, though it can be extended to other mating designs with binary genotypes (e.g. backcross, double-haploid). Clearly, our method is applicable to a wider set of designs and it does not require the phenotype data in order to compute a probability for missing genotype. In contrast to others [8], the recombination rate is not estimated separately but rather a specific parameter is computed within the algorithm that plays a similar role. Our imputation method considers two separate models, one for markers at the edge of a chromosome and another for all others. Each model requires an estimation of a recombination rate parameter. The model-specific parameter for middle markers is estimated as a function of the genotypes of the two flanking markers and the genetic distances towards those neighbors. A distinguishing characteristic of our imputation method is that the outcomes are probabilities which correspond to weights of observing one of the two parental lines at that locus. We integrate these weights into a lasso [10] to advance the QTL identification. The proposed analysis pipeline is validated using extensive simulations and compared to alternative methods. It is then applied to a real dataset that motivated our method and which is described next.

## Motivating example

The primary biological goal of this work is QTL detection and the eventual goal is to improve the quality of seed production in Arabidopsis thaliana. It has been shown that measurements of the germination rate of maize in the laboratory could predict the relative performance in field sowing [11]. An increase in sowing performance can result in economically important crops. A similar strategy is taken in our study where germination characteristics of Arabidopsis seeds were examined in order to find QTLs associated with each trait [12]. Lines from recombinant inbred population are important and convenient resources to study the genetic mapping of quantitative traits in plants or animals.

**Figure 1:**
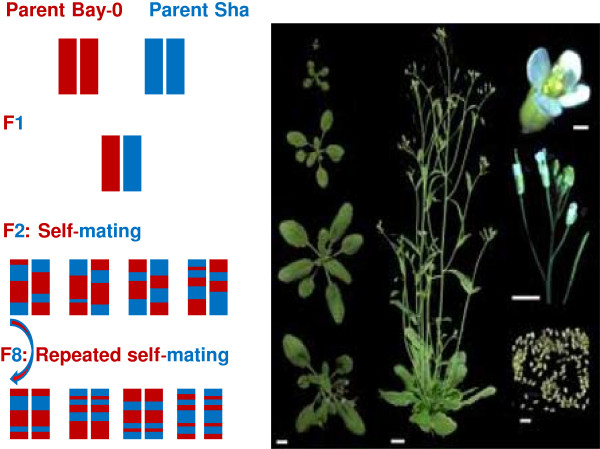
**Arabidopsis RIL procedure.** Left-RIL procedure with self-mating over 8 generations; right-Arabidopsis plant (source: http://www-ijpb.versailles.inra.fr/), namely left, the vegetative stage, before flowering and growth of the floral stalk (bottom left). On the center an adult plant at full flowering/seed set. On the rigth, flower, floral stem and seeds. White bars represent 1 cm, except for flower and seeds: 1 mm.

All RILs have the same parents. Each RIL has a unique combination of loci derived by recombination of the alleles present in the parents. Thus, each RIL has a unique genetic make-up. One traditional way of RIL construction is to cross two parental plants to produce an F1 generation, followed by several consecutive generations of self-mating. This results in a so called “core population”. These lines are practically homozygous and can be propagated indefinitely as clones. Biological and technical details of the RIL procedure are shown in Figure 1.

In our study, F8 seeds from 165 lines of Arabidopsis were obtained from the Versailles Biological Resource Centre [13]. The seeds were the results of cross between Bayreuth and Shahdara Arabidopsis plants, using an inbreeding approach over eight generations. Bay-0 originates from a fallow-land habitat near Bayreuth in Germany, whereas Shahdara grows at high altitude in the Pamiro-Alay mountains in Tadjikistan [14]. The Bay-0 and Sha RIL populations have been used in several previous studies to map QTLs [12]. Arabidopsis has five chromosomes. For every RIL, 69 markers were genotyped with an average genetic distance of 6.1 centimorgans (cM) between markers [14]. Of the 69 included markers, respectively 18, 11, 12, 11 and 17 markers are located on chromosomes 1, 2, 3, 4 and 5. The lengths of chromosomes are 91.3, 64.6, 72.2, 69.1 and 91.2 cM respectively.

### Arabidopsis germination experiment

The phenotyping experiment was conducted in two stages: (1) seed sowing followed by measurements of grown plant traits and (2) collecting seeds from these plants followed by germination [12]. In this study we examine traits from the second part of the experiment, namely germination. In the first stage of the experiment (2008), Arabidopsis seeds obtained from Versailles were randomly allocated to three plates and grown in a climatized chamber. The plates can be considered technical replicates, each with 3-5 RIL plants. One year later, seeds stored in 2008, were planted on a fourth plate. In addition, the best seeds (free of fungus, etc.) from the first three plates were collected and germinated in 2009 on a fifth plate. In the second stage of the experiment, 50-100 seeds from each line of the core population of grown plants were collected and germinated.

Several factors were varied or simply needed to be accounted for in germination experiment, such as seed age, dormancy, imbibition, growing plate (and selection), temperature, and chemical stress. With respect to dormancy, i.e. storage conditions, two types can be identified, namely fresh and after-ripened (AR) [12]. Besides normal temperature, two types of temperature stresses were applied, namely cold and heat shock. The following chemical stresses were applied: table salt, osmotic-inducing mannitol, oxidizer hydrogen peroxide, inhibitor abscisic acid (ABA), and controlled deterioration (CD) [12]. Germination process under all chemicals except hydrogen peroxide was carried out in light and dark imbibition conditions.

**Figure 2:**
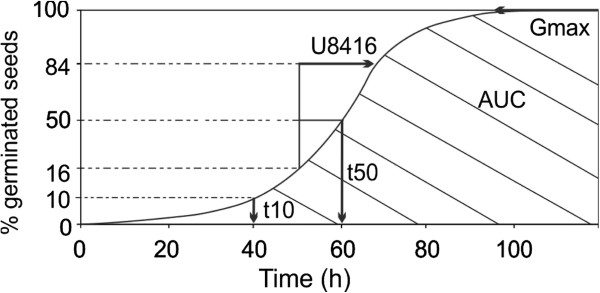
**Cumulative germination curve, described by G**_**max**_** (%), T**_**50**_** (h), T**_**10**_** (h), U**_**8416**_** (h), and AUC**_**100**_** (% × h).**

Cumulative germination data were gathered to estimate the germination performance. Five relevant parameters from the germination-time curve were extracted using the Germinator package [13]. These parameters are (1) the percentage of maximum germination, G_max_, indicating the maximum germination capacity of a seed lot, (2) the time to reach 10% of germination, T_10_, indicating initiation of germination, (3) the time to reach 50% of germination, T_50_, indicating rate of germination, (4) the time between 16% and 84% of germination, U_8416_, indicating uniformity of germination, and (5) the area under the germination curve between t=0 and t=100 hours, AUC_100_ (see Figure 2).

### Statistical design

All five traits are continuous traits (G_max_ (%), T_50_ (h), T_10_ (h), U_8416_ (h), AUC_100_ (*%*× h)). Higher G_max_ means more germination. Lower T_10_ and T_50_ mean faster germination. The aim of this QTL analysis is to find the markers that are associated with these five response variables. We use the terms “(quantitive) trait” and “response variable” interchangeably.

The set of explanatory variables are the 69 markers. Our genetic dataset contains genotypes of 69 markers for 167 plants (including parental Bay and Sha). Marker genotypes for Bay and Sha are denoted by 0 and 1 respectively. Genotypes across all markers are the same for each parent. Genotypes in children plants are inherited from either of the parents, therefore each marker has only two possible genotypes {0,1}. So, technically, the 69 markers can be seen as a distinct combination of 0’s and 1’s across the RILs.

All 167 plants are treated under 42 different conditions resulting in 7014 observations for every trait, some of which are missing. These conditions are made up from combination of the factors (described in previous section), which are not of primary interest, but which must be taken into account. Conditions are age, dormancy (Fresh, AR), plate (1-5), imbibition (light, dark), temperature (8, 10, 20, 25, 30 degrees Celcius) and chemical stress (no, salt, mannitol, hydrogen peroxide, ABA, CD). Hence, each response variable is adjusted for these nuisance variables. Such adjustment confirms that any detected marker effect is robust under all these conditions.

### Missing data

The phenotypes G_max_, T_50_, T_10_, U_8416_, and AUC_100_ contain respectively 0.49*%*, 1.90*%*, 1.90*%*, 1.92*%* and 0.49*%* missing data. It seems reasonable to assume that the missingness of any observation for a given trait is independent of the observed and unobserved values. Such missing mechanism is known as missing completely at random (MCAR). Furthermore, the small percentage of missingness across all phenotypes means that we can safely omit the missing observations from our analysis, even if the MCAR assumption is not true.

We summarized the number of missing markers per plant and the number of missing plants per marker. About 25% of RILs do not have missing values for any marker. The remaining RILs have up to 9 missing values, with only two RILs have 20 and 24 missing markers. As for the number of missing plants per marker, we counted that each marker has at least one missing RIL. The number of missing RILs per marker vary between 1 and 13. Missingness in markers may be caused by essay quality, poor hybridization and/or other reasons. Such nature of missingness is unlikely to be MCAR, as the missingness may well statistically depend on whether its neighbor is missing – due to the sequential operation of the genotyping instrument. This missingness feature might then be described as missing at random (MAR) since the missingness does not depend on the unobserved markers themselves, but it does depend on the observed markers, i.e. we observe whether its neighbor is missing or not and this enables prediction of the probability that this marker is missing.

## Methods

### Marker probability model

There are only two possible genotypes for each marker in a RIL experiment. Let $x_{c,t}^{\left(i\right)}$ be the parental type for a RIL *i* at chromosome *c* at genetic location *t*. Namely:

(1) $$x_{c,t}^{\left(i\right)}=\left\{\begin{array}{cc} 0,\; & \text{if parental type at}(c,t)\text{for RIL}\;i\;\text{is Bay}\\ 1,\; & \text{if parental type at}(c,t)\text{for RIL}\;i\;\text{is Sha.} \end{array}\right)$$

We assume that for each marker the genotype at position *t* depends on the genotypes of two immediate neighboring markers at positions *t*_0_ and *t*_1_, as well as the distances (*t*_1_−*t*) and (*t*−*t*_0_) assuming *t*_0_<*t*<*t*_1_. It would be reasonable to assume an Ising model on switching the genotypes from one marker to another:

(2) $$P\left(x_{c,t}^{\left(i\right)}|{\left\{x_{c,s}^{\left(i\right)}\right\}}_{s\leq t_{0}\cup s\geq t_{1}}\right)=P\left(x_{c,t}^{\left(i\right)}|x_{c,t_{0}}^{\left(i\right)}x_{c,t_{1}}^{\left(i\right)}\right),$$

where *t*_0_ and *t*_1_ are genetic locations of flanking markers and $x_{c,t_{0}}^{\left(i\right)}$ and $x_{c,t_{1}}^{\left(i\right)}$ are genotypes of those markers. In our case genetic locations *t*_0_, *t* and *t*_1_ refer to genetic distances from starting point of a chromosome.

As stated above, markers in a RIL have only two genotypes {0,1}. There are two possible sources of the genetic variability, genetic recombination and mutation. Recombination/meiosis is a process of chromosomal crossover whereby two chromatids can mesh with one another. The variations, isolated by breeders are the result of recombination and not mutation due to short period of time involved with the isolation of the varieties. Variation due to mutation on the time-scale of this experiment (i.e. 6/8 generations) is dwarfed by the variation of recombination.

**Figure 3:**
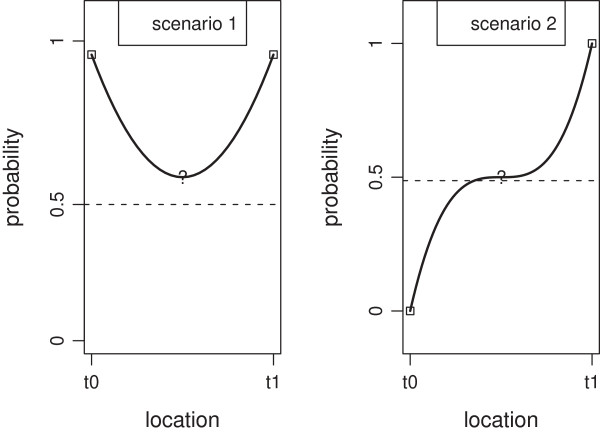
**Possible scenarios for genotypes of three consecutive markers located at locations** ***t***_**0**_**,** ***t* (at mark ?) and** ***t***_**1**_** for RIL** ***i*: (scenario 1) y axis shows the probability of observing Sha at location** ***t* given we observe Sha at locations** ***t***_**0**_** and** ***t***_**1**_**,**$P(x_{c,t}=1|x_{c,t_{0}}=1,x_{c,t_{1}}=1)$**; (scenario 2) y axis shows the probability of observing Sha at location** ***t* given we observe Bay at** ***t***_**0**_** and Sha at** ***t***_**1**_**,**$P(x_{c,t}=1|x_{c,t_{0}}=0,x_{c,t_{1}}=1)$**.**

This physical intertwining during meiosis induces naturally (though not necessarily) a Markov dependence structure, whereby knowledge of the configuration of chromatids at a particular marker depends only on the neighboring configurations. With respect to the shape of the dependence structure, we note that it is natural that the absolute value of the derivative of the probability model is minimal exactly half-way through the interval between two known markers. This reflects the fact that the amount of change of information is smallest when we are further away from the known markers. This leads to four possible scenarios of a marker with immediate neighbors. Figure 3 shows two of such scenarios, the remaining two are symmetric (about the x-axis) of the ones depicted on this Figure. These scenarios are meaningful only for markers having both neighbors. In turn, a marker has both neighbors if it is not at the edge of a chromosome. As for edge markers, only two scenarios are possible (not depicted here). Considering scenarios given in Figure 3, we propose a model that exhibits the same shape. We introduce a parameter *α*∈[0;*∞*) which technically has a scaling function and biologically it has a role similar to the recombination rate. The probability model for a RIL *i* and a marker located in the middle of a chromosome is: 

where *x*_0_,*x*_1_∈{0,1} and *δ*_*x*_ is defined by:

(4) $$\delta_{x}=\left\{\begin{array}{cc} 1,\; & \text{if}\;x=1\\ -1,\; & \text{if}\;x=0. \end{array}\right)$$

Note, for a marker surrounded by two known markers, if *α*=0 this means the recombination rate is zero. Therefore, (3) gives us that the probability of a Sha marker between two given Sha markers is 1. In contrast, if *α*→*∞* then the recombination rate is infinite meaning that there is no information in neighboring markers. Therefore, (3) gives us a probability of 0.5 for any value of the flanking markers.

Likewise, for markers at right edge of a chromosome the probability model involves a parameter *β*∈[0;*∞*):

(5) $$\pi (\beta )\equiv P\left(x_{c,t}^{\left(i\right)}=1|x_{c,t_{0}}^{\left(i\right)}=x_{0}\right)=\frac{1}{2}+\frac{1}{2}\delta_{x_{0}}\beta^{\left(t-t_{0}\right)},$$

where *x*_0_∈{0,1} and *δ*_*x*_ is defined as in (4). The probability model for the markers at left edge of a chromosome is similar to equation (5).

### Pseudo maximum likelihood in imputation

To estimate the parameters *α* and *β* in probability models (3) and (5) we use only the children plants and not the parents. The pseudo log-likelihood [15,16] of a model (3) for middle markers is:

(6) $$\begin{array}{ll} \ell (\alpha ) & ≅\Sigma_{i,c,t}\ln P\left(\overset{\left(i\right)}{\underset{c,t}{x}}|x_{c,(t-1)}^{\left(i\right)},x_{c,(t+1)}^{\left(i\right)}\right)\\ & =\Sigma_{i,c,t}\left[x_{c,t}^{\left(i\right)}\ln(\pi (\alpha ))\right)\\ & \;\left(+\left(1-x_{c,t}^{\left(i\right)}\right)\ln(1-\pi (\alpha ))\right], \end{array}$$

from which we estimate *α*. In a similar way we estimate *β*.

We examined whether separate parameters are required for every chromosome. For middle markers we tested *H*_0_: *α*_1_=*α*_2_=*α*_3_=*α*_4_=*α*_5_ versus *H*_1_: at least one *α* differs from others. Subsequently, we computed the pseudo log-likelihood on five parameters and compared it with the pseudo log-likelihood on one parameter using the pseudo likelihood ratio test (LRT) statistic:

(7) $$\text{LRT}=-2[\ell ({\hat{\alpha }}_{\text{OneChr}})-\ell ({\hat{\alpha }}_{\text{FiveChr}})].$$

The distribution of the LRT is the weighted sum of four independent $\chi_{1}^{2}$ distributions [17]. Since the pseudo likelihood is probabilistically close to the true likelihood, the LRT can also be approximated by $\chi_{4}^{2}$. Alternatively, the p-value can be calculated using a bootstrap approach.

We also tested the goodness of fit of the proposed model using Pearson’s chi-squared statistic.

### Genotype imputation

The missing markers are substituted with a one or a zero, by rounding the probability $P(x_{c,t}^{\left(i\right)}|x_{c,t_{0}}^{\left(i\right)}x_{c,t_{1}}^{\left(i\right)})$ to an integer. The substituted value is more certain when the probability is closer to zero or one. It is less certain when the probability is close to 0.5. In the analysis we will use the weights that would represent this uncertainty. The weights for the missing genotypes are:

(8) $$w_{c,t}^{\left(i\right)}=2|P\left(x_{c,t}^{\left(i\right)}|x_{c,t_{0}}^{\left(i\right)}x_{c,t_{1}}^{\left(i\right)}\right)-\frac{1}{2}|,$$

where $w_{c,t}^{\left(i\right)}\in [0,1]$. Indeed, zero weights are used for genotypes with rounded probabilities equal to 0.5 and weights of ones are used when the imputed probabilities approach to zero or one. For non-missing genotypes, we assume that they are observed with complete certainty resulting in a weight of one. The highest possible weights are given to the observed genotypes. The weights $w_{c,t}^{\left(i\right)}$ are computed from the imputed (predicted) probabilities $\hat{P}(x_{c,t}^{\left(i\right)})$. These probabilities were estimated via the maximum likelihood estimation procedure given in previous two sections.

### Phenotype response

We adjusted every trait for the effect of the nuisance variables (age, dormancy, plate, imbibition, temperature, chemical, Bay, Sha). For observation *i*, the adjustment was carried out using a linear regression as shown on the example of G_max_ below:

(9) $${\text{Gmax}}_{i}=\phi_{0}+\phi_{1}{\text{age}}_{i}+\ldots \phi_{m}{\text{Sha}}_{i}+y_{i},$$

where the residual variance is constant across all observations and the residual is distributed independently and identically (iid), *y*_*i*_∼*iidN*(0,*τ*^2^). The model (9) employs *m* regression parameters *ϕ*_0_,…,*ϕ*_*m*_. Then we computed the difference between the observed and fitted values for every observation and used these residuals *y*_*i*_ as inputs to weighted lasso.

### Weighted lasso phenotype inference

The original lasso weights all observations equally [10]. An adaptive lasso is an extension of lasso by weighting or penalizing different coefficient differently in a way that depends on the data [18]. The proposed weighted lasso (wlasso) is a different extension of the lasso by weighting different observations differently in a way that depends on the data and the value of the coefficients. An adaptive lasso places weights in the penalty part of the objective function, whereas wlasso places weights in the sum of squares part of the objective function.

For convenience, we use in this section the notation *x*_*ij*_ and *w*_*ij*_ instead of $x_{c_{j},t_{j}}^{\left(i\right)}$ and $w_{c_{j},t_{j}}^{\left(i\right)}$ for the genotype and weight information of RIL *i* at chromosome *c*_*j*_ at location *t*_*j*_, since information about chromosomal locations and genetic distances have already been incorporated into the estimates of the probabilities *π*(*α*) or *π*(*β*) and the corresponding weights. Below we describe the wlasso algorithm. Let ***X*** be the matrix that contains original genotype values {0,1} and the rounded imputed probabilities; *x*_*ij*_ is an element of this matrix for RIL *i* and marker *j*. *y*_*i*_ is the residual response for RIL *i*, as described in previous section. We assume that the observations are independent. We define the wlasso estimate $\hat{\theta }$ as:

(10) $$\begin{array}{ll} {\hat{\theta }}_{\lambda } & ={\mathrm{argmin}}_{\theta }\left\{\overset{n}{\underset{i=1}{\sum }}\frac{\overset{p}{\underset{j=1}{\sum }}w_{\text{ij}}|\theta_{j}|}{\overset{p}{\underset{j=1}{\sum }}|\theta_{j}|}{\left(y_{i}-\overset{p}{\underset{j=1}{\sum }}\theta_{j}x_{\text{ij}}\right)}^{2}\right)\\ & \;\left(+\lambda \overset{p}{\underset{j=1}{\sum }}|\theta_{j}|\right\}. \end{array}$$

The idea behind the method is to downweight observations with a lot of imputed values *x*_*ij*_: note for instance that observations with all weights *w*_*ij*_ zero are eliminated from the regression, whereas observations with a fully observed *x*_*i*._, and therefore all weights *w*_*ij*_ equal to one, are fully taken into consideration. Moreover, observations that have only missing values on marker locations *j* that are deemed irrelevant for the regression, i.e. ${\hat{\theta }}_{j}=0$, will not be penalized for their partial missingness. The model naturally accounts for imputation imprecision, without letting irrelevant imputations affects the quality of the estimate and with the ordinary lasso as limiting case when no imputation was performed.

Just as in ordinary lasso, the selection of “best” regularization parameter *λ* is not obvious. Various selection methods, such as the Bayesian information criterion (BIC), Akaike information criterion (AIC) and cross-validation, have been proposed. As suggested [19], the BIC is the most relevant criterion when the sparsity of the model is of primary concern. The BIC tends to lead to consistent selection of *λ* and quite sparse models with relevant biological interpretation. Therefore it is used in this study, i.e. we minimize the following objective function across *λ*:

(11) $$\text{BIC}(\lambda )=\overset{n}{\underset{i=1}{\sum }}\frac{{\left(y_{i}-\overset{p}{\underset{j=1}{\sum }}{\hat{\theta }}_{\lambda ,j}x_{\text{ij}}\right)}^{2}}{s^{2}}+\hat{\text{df}}(\lambda )\ln(n),$$

where *s*^2^ is some robust estimate of the variance that does not depend on *λ* and $\text{df}(\lambda )=\overset{p}{\underset{j=1}{\sum }}1_{\left\{{\hat{\theta }}_{\lambda ,j}\neq 0\right\}}$ is the number of non-zero parameters in the model.

Given that (10) cannot be minimized explicitly, we use an iterative procedure to obtain ${\hat{\theta }}_{\lambda }$, which given initial non-zero weights is guaranteed to converge to the global minimum. In practice, we define initial estimates ${\hat{\theta }}^{\left(0\right)}=({\hat{\theta }}_{1}^{\left(0\right)},{\hat{\theta }}_{2}^{\left(0\right)},\ldots ,{\hat{\theta }}_{p}^{\left(0\right)})$ for all markers using the regular lasso. Such initialization is the same as assigning weights of ones $w_{i}^{\left(0\right)}=1$ to every RIL *i*. In iteration *k*+1, the regression parameters are updated as:

(12) $$\begin{array}{ll} {\hat{\theta }}^{\left(k+1\right)} & ={\mathrm{argmin}}_{\theta }\left\{\overset{n}{\underset{i=1}{\sum }}w_{i}^{\left(k\right)}{\left(y_{i}-\overset{p}{\underset{j=1}{\sum }}\theta_{j}x_{\text{ij}}\right)}^{2}\right)\\ & \;\left(+\lambda \overset{p}{\underset{j=1}{\sum }}|\theta_{j}|\right\}, \end{array}$$

where the weights $w_{i}^{\left(k\right)}$ for each RIL in equation (12) are updated in an iterative way as:

(13) $$w_{i}^{\left(k\right)}=\frac{\overset{p}{\underset{j=1}{\sum }}w_{\text{ij}}|{\hat{\theta }}_{j}^{\left(k\right)}|}{\overset{p}{\underset{j=1}{\sum }}|{\hat{\theta }}_{j}^{\left(k\right)}|}.$$

We are defining convergence as the first *k*, such that $|w_{i}^{\left(k+1\right)}-w_{i}^{\left(k\right)}{|}^{2}<\varepsilon$, where *ε* is a predefined tolerance level. Using tolerance level *ε*=10^−8^ all five traits converged in 4 iterations. The plots of weights are included for visualization convenience (see Additional files 1, 2 and 3).

## Results

In this section, we first present the results of detecting QTL effects for germination in Arabidopsis. Then, we present the strategies and results from three simulation studies. With the first simulation study we aim to justify our proposed methodology. In the second and third simulation studies we compare our methodology with alternative methodologies for the case study. The second study emphasizes a comparison of our imputation methods with the nearest marker imputation. The third study focuses on comparison of sparse variable selection techniques, namely our weighted lasso, the traditional lasso [10] and adaptive lasso [18].

### Analysis of Arabidopsis germination experiment

**Figure 4:**
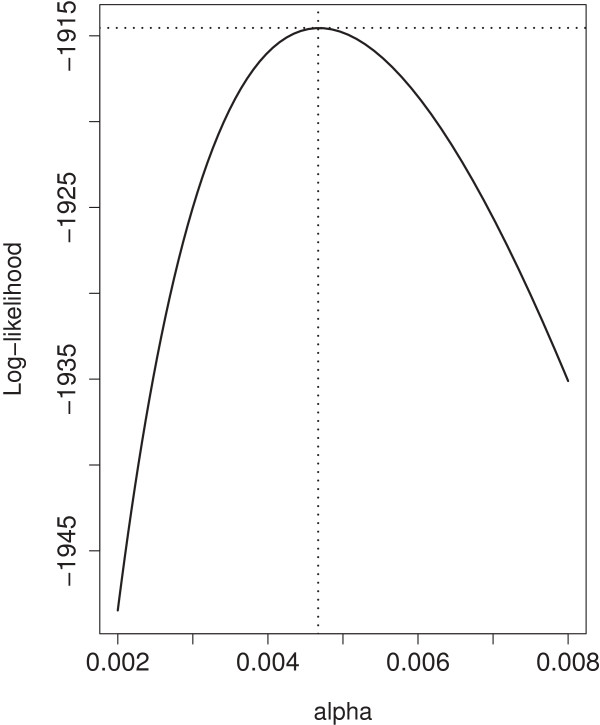
**Plot of log-likelihood for middle markers with maximum at (**$\hat{\alpha }=0.0047$**,**$\hat{\ell }=-1914.542$**).**

**Figure 5:**
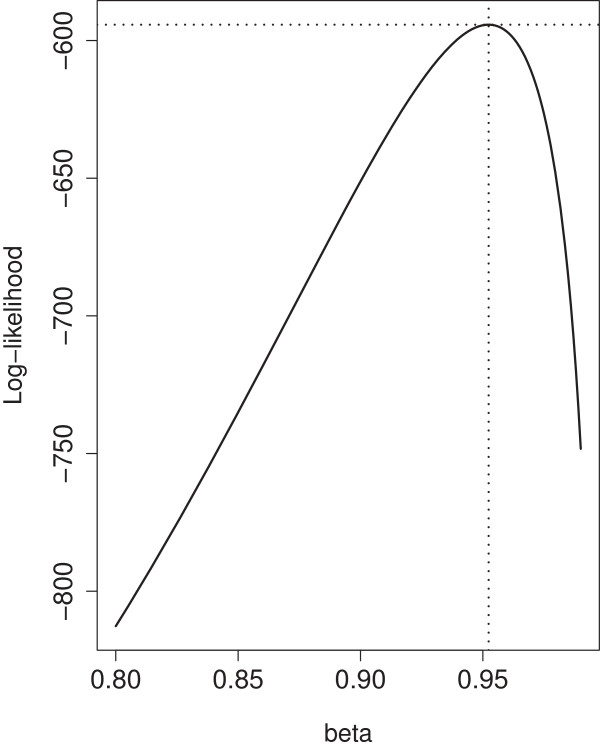
**Plot of log-likelihood for edge markers with maximum at (**$\hat{\beta }=0.9524$**,**$\hat{\ell }=-594.24$**).**

For the genotype data, we estimated two recombination rate parameters, depending on whether the marker was on the edge or in the interior of a chromosome. The parameters *α* and *β* in probability models (3) and (5) were estimated using 165 children plants. The maximum pseudo log-likelihood estimates for these parameters were $\hat{\alpha }=0.0047$ and $\hat{\beta }=0.9524$ (see Figures 4 and 5). The need for introducing a separate parameter for each chromosome is shown not to be necessary for interior markers (LRT=4.633, df=4, chi-square p-value=0.327, bootstrap p-value=0.456) and for edge markers (LRT=4.116, df=4, chi-square p-value=0.391). The goodness of fit of the proposed recombination models for interior and edge markers was tested using Pearson’s chi-squared statistic. The results suggest a good fit of both models (for interior marker: *χ*^2^=7458.23, df=9280, p-value=1; for edge markers: *χ*^2^=1210.51, df=1591, p-value=1), suggesting that we do not need more than just the flanking markers to infer the genotype of the missing marker. This is in agreement with the traditional meiosis model of recombination.

**Figure 6:**
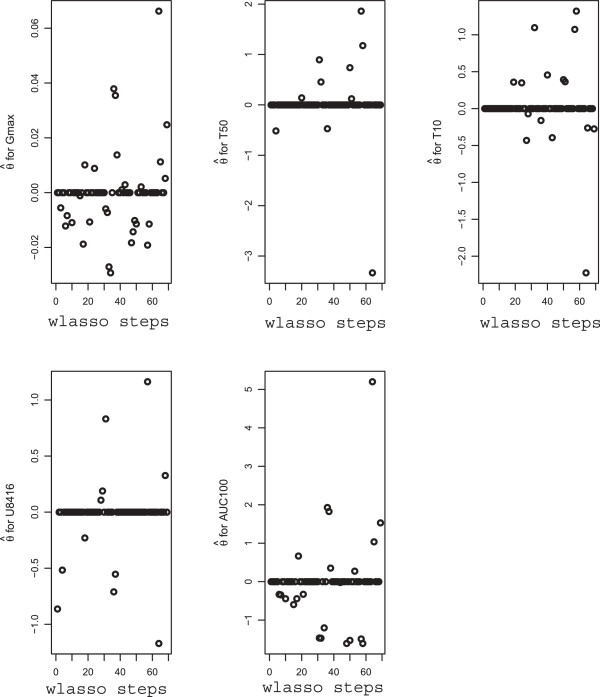
**Plots of weighted lasso regression coefficients**$\hat{\theta }$** vs. number of steps in weighted lasso for traits G**_**max**_**, T**_**50**_**, T**_**10**_**, U**_**8416**_**, and AUC**_**100**_**.**

**Figure 7:**
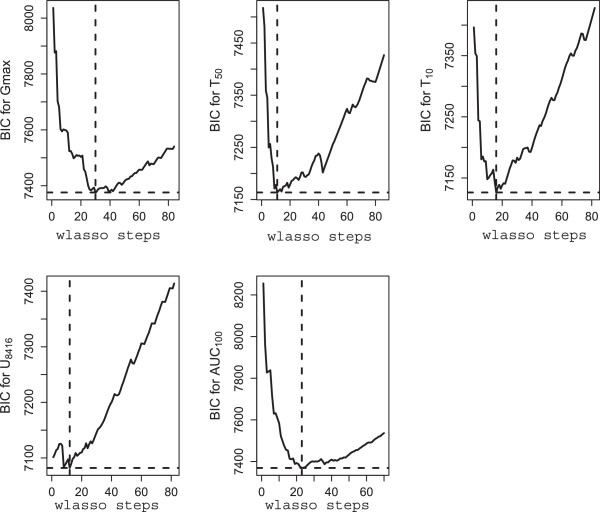
**Plots of BIC vs. number of steps in weighted lasso for traits G**_**max**_**, T**_**50**_**, T**_**10**_**, U**_**8416**_**, and AUC**_**100**_**.**

We adjusted all 7014 observations of every trait using regression model (9). Number of regression parameters *ϕ*_0_,…,*ϕ*_*m*_ is high since some of the input variables are categorical. In total we estimated *m*=15 parameters. Then, the residuals were modeled using wlasso, as described in previous sections. The marker effects, demonstrated by regression coefficients in wlasso, are presented in Figure 6. These values will be used in the simulation studies below. The BICs for all five traits are shown in Figure 7. For each trait, those markers are selected by wlasso as indicated by the minimal BIC value. Thus, on the basis of BIC 29, 10, 15, 11 and 22 markers are selected for G_max_, T_50_, T_10_, U_8416_, and AUC_100_ respectively. Clearly, the number and selection of markers differ for each trait. They represent in total 39 out of the 69 available markers.

**Figure 8:**
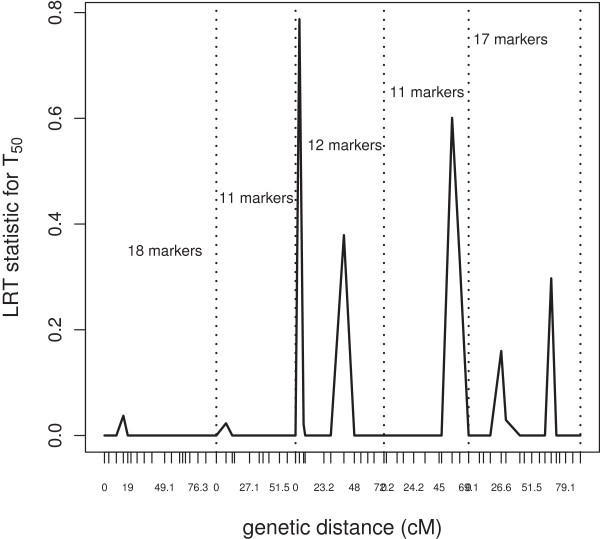
**Plot of LRT statistic (on log(10) scale) of markers associated with T**_**50**_** vs. genetic distance; dashed lines separate 5 chromosomes.**

We computed the LRT under the full and a restricted models on a 10-base logarithm scale for markers selected by wlasso. We present the 39 marker names, genetic distances and respective LRT statistic in Table 1. The larger the LRT statistic, the stronger the evidence in favor of a QTL effect at a particular location of the chromosome. The LRT statistic below 0.01 was substituted by 0. We would like to emphasize that detecting peaks that contain several markers increased our confidence in the region being associated with a trait. For example, the beginning of chromosome 3 (with markers MSAT399, ATHCHIB2, MSAT305754) and middle of chromosome 4 (MSAT415, CIW7, MSAT418) give strong evidences of these regions to be associated with G_max_ (we see a set of genes with a high LRT statistic). Loci at chromosomes 3 and 5 are strongly indicative for all five traits. We also visualized the LRT statistic across genetic distance and presented the plot for T_50_ in Figure 8. Five peaks with eight markers are seen for T_50_ peak_1_-MSAT399, ATHCHIB2; peak_2_-MSAT332; peak_3_-MSAT49, MSAT468; peak_4_-MSAT514, NGA139; peak_5_-MSAT520037. We have the highest confidence in peak_3_ since it contains multiple markers having the large LRT statistic. All peaks except the one at chromosome 4 have been detected by biologists as well [12]. Thus, we have found one additional peak for T_50_.

**Table 1:** **LRT statistic of markers selected by weighted lasso for G**_**max**_**, T**_**50**_**, T**_**10**_**, U**_**8416**_**, and AUC**_**100**_** ordered by genetic distance across 5 chromosomes in Arabidopsis**

**Marker**	**chr**	**gdist**	***G***_***max***_	***T***_**50**_	***T***_**10**_	***U***_**8416**_	***AUC***_**100**_
MSAT100008	1	0				0.64	
F21M12	1	9.7	0.09				
IND4992	1	15.4		0.04		0.32	
MSAT110	1	21.6	0.02				0
MSAT108193	1	26.6	0.14				0.21
T27K12	1	49.1	1.27				0.65
F5I14	1	69.6	0				0
MSAT127088	1	82.7	0.15				0.33
MSAT15	1	91.3	0.01			0.81	0
MSAT25	2				0.19		
MSAT200897	2	7.9		0.02			
MSAT238	2	13	0.16				0.15
MSAT241	2	35	0.09		0		
IND216199	2	51.5			0.03		
MSAT210	2	57.9			0.17	0.24	
MSAT222	2	64.6				0.86	
MSAT399	3	3.2	0.68	0.79		0.58	1.03
ATHCHIB2	3	6.6	0.57	0.02	0		0.16
MSAT305754	3	7.9	0.58				
MSAT319	3	23.2	0.03				0.13
MSAT332	3	39.5	0.17	0.38	0.51	0.03	0.31
MSAT321	3	48	0.33			0.09	0.25
MSAT318406	3	53.3	0.05				0
MSAT318	3	64.1			0		
MSAT370	3	72.2	1.28				
MSAT48	4	2	0.09		0.04		
MSAT443	4	10.7					0.52
MSAT415	4	33.5	0.45				
CIW7	4	45	1.22				0.29
MSAT418	4	47	0.03				
MSAT49	4	55.6	0.06	0.60	0.31		0
MSAT468	4	61.8		0.33	0.17		
MSAT500027	5	0	0.34				0.5
MSAT514	5	26.6	0	0.16	0.01	0.46	0.09
NGA139	5	30.4	0.42	0.03	0		0.13
MSAT520037	5	67.4	0.9	0.30	0.27	0.01	1.07
MSAT512	5	71.6	0.13		0.39		0.31
MSAT519	5	85	0.26			0.01	
K9I9	5	91.2	0.35		0.08		0.25

None of the detected peaks at chromosomes 2 and 4 were identified before [12]. Peaks at chromosome 2 are relatively low. The region with MSAT25 provides relatively high confidence for association with T_10_. The duo markers (MSAT238 and MSAT241, IND216199 and MSAT210, MSAT210 and MSAT222), with a considerable LRT value, demonstrate a certain confidence of association with G_max_, T_10_ and U_8416_ respectively. Thus, regions at chromosome 2 should be considered among QTL despite their low LRT statistic. Similar interpretations apply to detected regions at chromosome 4. In addition, several peaks detected by us at chromosomes 1, 3 and 5 have not been identified by others [12].

**Figure 9:**
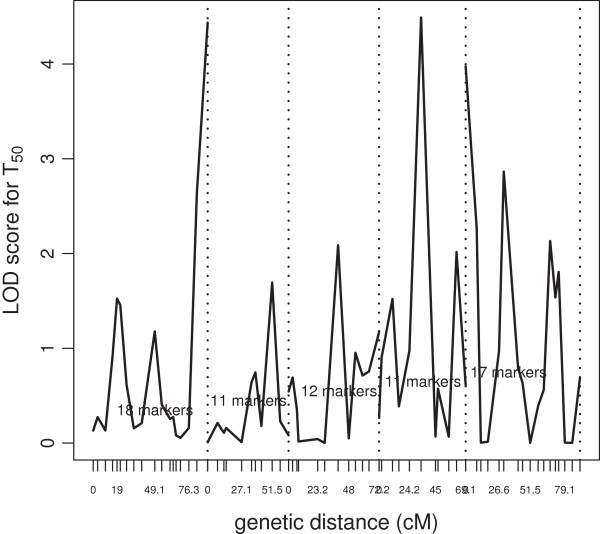
**LOD score for T**_**50**_** vs. genetic distance; dashed lines separate 5 chromosomes.**

For comparison with LRT given above, we computed the LOD-score for every marker across each trait. The LOD-score is essentially the LRT statistic on a 10-base logarithm scale. It measures the strength of evidence for the presence of a QTL at a particular location. The LOD-score for each marker is computed as the LRT under the full and a restricted models. The full model includes all 69 markers, while the restricted model includes all but the marker of interest. We present the LOD-scores for T_50_ in Figure 9 and it is clear that some of the peaks from the LRT are much sparser and that several large peaks have disappeared. This shows the instability of the ordinary multiple regression approach as compared to the stable lasso method.

### Simulation strategies

Simulating genotype data by an Ising model (interaction parameter *η*) is quite realistic for a recombination process on a chromosome [20]. The parameter *η* shows the strength of the dependence between markers. We considered that inheritance at loci on different chromosomes are independent events and simulated the markers of every chromosome one at a time. We included the dependence between markers in two ways: (1) the dependence between equally-spaced neighboring markers using an Ising model with *η*=0.4, (2) the genetic distances between the observed markers by subsampling the full process. In particular, we rounded the genetic distance of every chromosome and simulated markers with genotypes {0,1} 1 cM apart for 165 RILs. Then we selected those markers which were spaced with the same genetic distances as markers in our RIL experimental data. Markers for every chromosome were simulated independently and then joined together as a genotype dataset.

We assumed that among all observed markers about 10% have the true QTL effect. Thus, the largest positive and negative $\hat{\theta }$ of 6 markers from weighted lasso of our real experiments were selected as the true effects (see Figure 6). Among the simulated 69 markers, 6 evenly spaced markers (along five chromosomes) were selected as the true input variables. An additive effect of markers was assumed and the response variable was generated using the multiple regression model. Residual error, having the normal distribution with mean *μ*=0, was added to the trait. We studied our method under several values of residual error variances, namely *σ*^2^=0.5,1,2,3. We also investigated our methodology with 6 true markers being clustered (3 markers on the first chromosome and 3 on the second one) and compared it with the case of evenly-spaced markers.

We studied two missing mechanisms among markers: (1) an MCAR using Bernoulli missingness and (2) MAR using an Ising missingness model. Following our experimental data, we explored the case with 10% of missingness. Thus, the probability parameter in Bernoulli distribution is 0.1. We assumed the stronger dependence *η*_*MAR*_=0.6 among missing markers than simply among observed and non-observed markers (*η*=0.4). For every above described scenario, we carried out 50 simulations. The simulated data were analyzed using the proposed imputation model and wlasso as well as alternative approaches. For every simulation scenario, we summarized the performance of the tested methodology using the receiver operating curve (ROC). For that we measured the fraction of true positives out of all positives, so called true positive rate (TPR) and the fraction of false positives out of the negatives, so called false positive rate (FPR). To be specific, TPR=TP/(TP+FN) and FPR=FP/(FP+TN), where TP, TN, FP, and FN are the numbers of true positives, true negatives, false positives, and false negatives. TPR and FPR are also known as the sensitivity and (1-specificity) respectively.

For the sparse variable selection methods, we studied the TPR and FPR across a range of the lasso regularization parameter *λ* (BIC is not employed here as it was for real case study).

### Simulation study 1: justification of the proposed methodology

**Figure 10:**
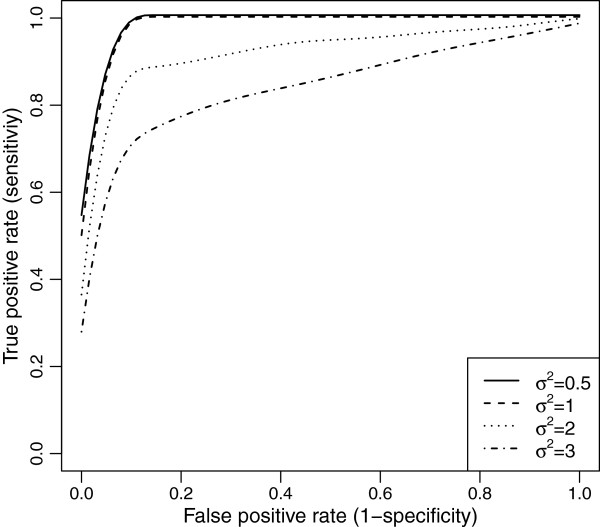
ROC curves comparison across different residual error variances for evenly-spaced markers with MCAR mechanism.

**Figure 11:**
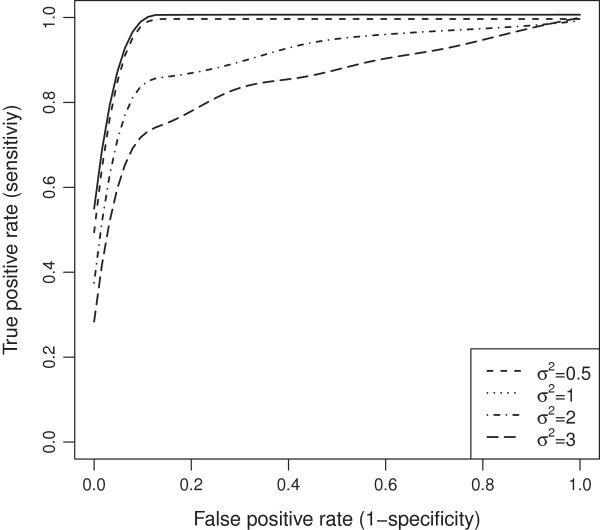
ROC curves comparison across different residual error variances for evenly-spaced markers with MAR mechanism.

**Figure 12:**
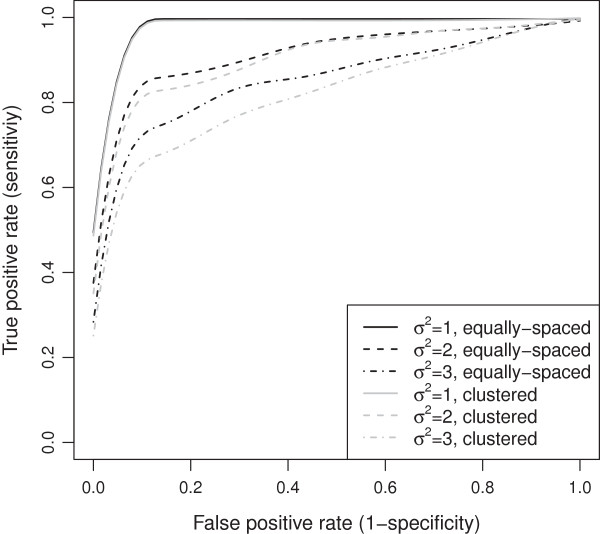
ROC curves comparison of evenly-spaced and clustered markers across different residual error variances with MAR mechanism.

The ROC curves for equally-spaced markers with MCAR and MAR are demonstrated in Figures 10 and 11. We see, that as the residual error variance increases, both the TPR and FPR decrease. This trend was very similar for clustered markers with both missing mechanisms. Though, as the residual error variance increases (*σ*^2^>1), the TPR and FPR drop more for clustered markers than for equally-spaced markers in MAR (see Figure 12).

To evaluate the accuracy of RIL experiments, we have to examine the results from real data in light of the simulation results. Traits G_max_ and T_50_ were examined as examples. In the simulation studies: regression coefficients were *θ*=0.5 (and up), investigated residual error variances were *σ*^2^=0.5,1,2,3, and the number of RILs was 165. In experiment for T_50_: regression coefficients were *θ*=0.5 (and up), residual error variance was *σ*^2^=150, and the number of observations was *n*=165×42=7014. These are equivalent to simulations with 165 RILs and *σ*^2^≈3.5. In experiment for G_max_: *θ*=0.02 (and up), *σ*^2^=0.04, *n*=7014. These are equivalent to simulations with *θ*=0.5 (and up), 165 RILs, and *σ*^2^=0.04(0.5/0.02)^2^/42≈0.6. From these residual error variances, we can find the corresponding ROC curves for T_50_ and G_max_ (see Figure 10 or 11). Thus, the results of T_50_ experiment are less powerful given the overall low ROC curve (*σ*^2^=3). In contrast, the results of G_max_ are highly stable, given the overall high ROC curve (*σ*^2^=0.5).

### Simulation study 2: comparison of the proposed methodology focusing on imputation methods

**Figure 13:**
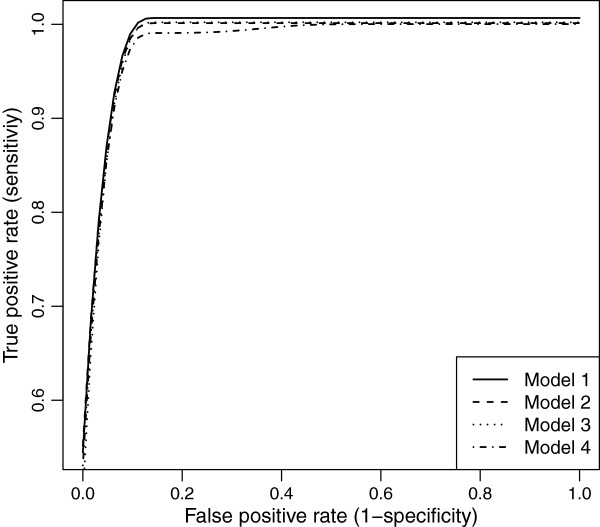
**ROC curves comparison across 4 models for clustered markers with MCAR mechanism when** ***σ***^**2**^**=0** ***.*** **5.**

**Figure 14:**
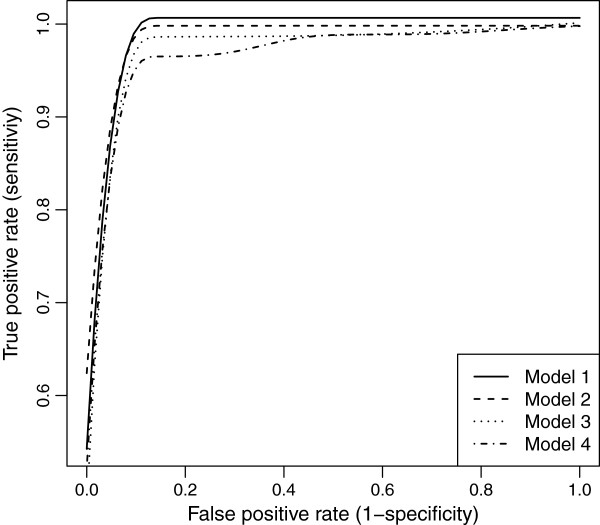
**ROC curves comparison across 4 models for clustered markers with MAR mechanism when** ***σ***^**2**^**= 0** ***.*** **5.**

**Figure 15:**
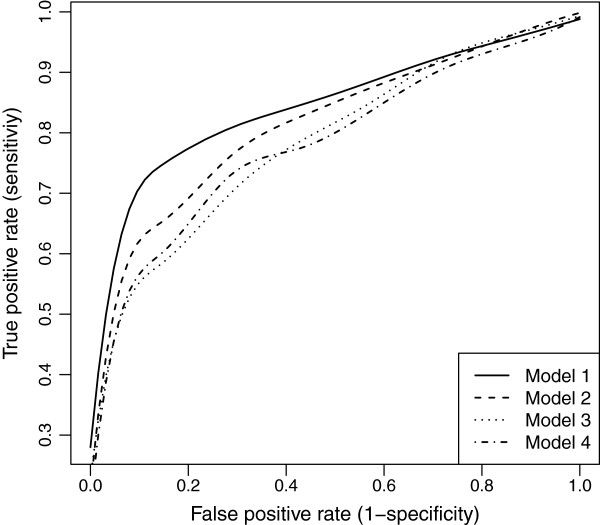
**ROC curves comparison across 4 models for evenly-spaced markers with MCAR mechanism when** ***σ***^**2**^**= 3.**

**Figure 16:**
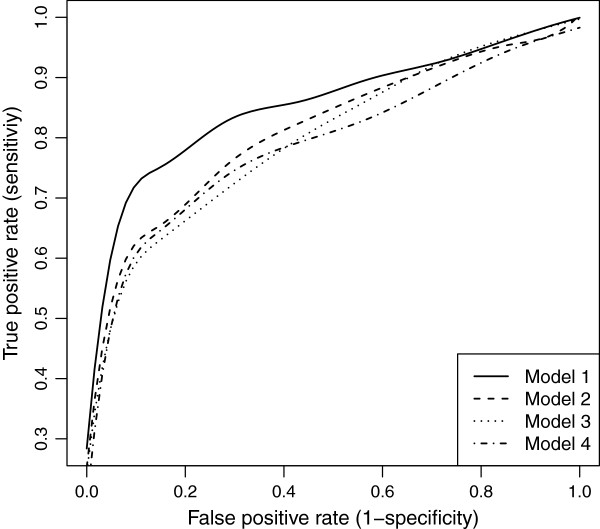
**ROC curves comparison across 4 models for evenly-spaced markers with MAR mechanism when** ***σ***^**2**^**= 3.**

**Figure 17:**
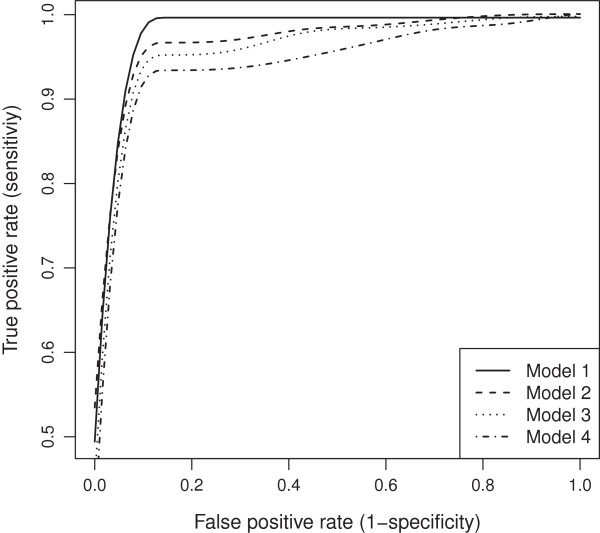
**ROC curves comparison across 4 models for evenly-spaced markers with MAR mechanism when** ***σ***^**2**^**= 1.**

**Figure 18:**
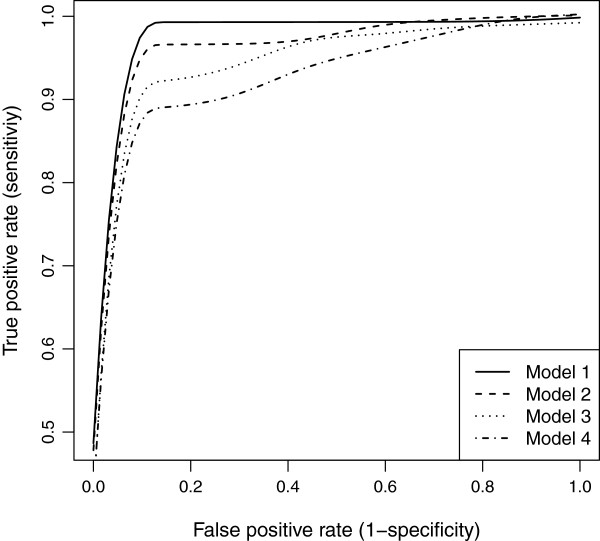
**ROC curves comparison across 4 models for clustered markers with MAR mechanism when** ***σ***^**2**^**= 1.**

The nearest marker imputation and multiple regression are frequently employed methods for QTL analysis. Our imputation was compared with the nearest marker imputation and wlasso was compared with the multiple regression. As a result we examined four models: (1) our imputation and wlasso, (2) our imputation and multiple regression, (3) nearest marker imputation and wlasso, (4) nearest marker imputation and multiple regression. Whenever wlasso was included in a simulation we studied the TPR and FPR across a range of the tuning parameter *λ*. Whenever multiple regression was included in a simulation we used the full significance level range [0,1] by increments of 0.015. Such increment results in 67 steps which are of the same order as number of steps in the wlasso. All four models were applied to simulated data described above and were studied for equally-spaced and clustered markers with MCAR and MAR mechanisms. The results were summarized using ROC curves. The ROC curves across four models for clustered markers when the residual error variance is small (*σ*^2^=0.5) are shown in Figures 13 and 14. Our model 1 outperforms others under all scenarios. We also show similar plots for larger residual error variance (*σ*^2^=3) when the markers are evenly-spaced (see Figures 15 and 16). Clearly, as the residual error variance increases, our Model 1 has more pronounced sensitivity and (1-specificity) than other models have. We also demonstrate the results for intermediate variance (*σ*^2^=1) when markers are evenly-spaced and clustered (see Figures 17 and 18). Interestingly, for smaller residual error variance (*σ*^2^≤1), the ROC curves of Model 2 are slightly above the curves of Model 3. This implies that the probabilistic imputation method with multiple regression has slightly higher accuracy than nearest marker imputation with wlasso. For larger residual error variance (*σ*^2^>1), the ROC curves of models 2 and 3 are approximately on top of each other, implying that the improvement for both wlasso and the probabilistic imputation method is roughly the same. However, just comparing our imputation method with nearest marker imputation for wlasso (Model 1 vs. Model 3) and for multiple regression (Model 2 vs. Model 4) demonstrates that our imputation method outperforms nearest marker imputation.

### Simulation study 3: comparison of the proposed methodology focusing on sparse variable selection techniques used for phenotype inference

**Figure 19:**
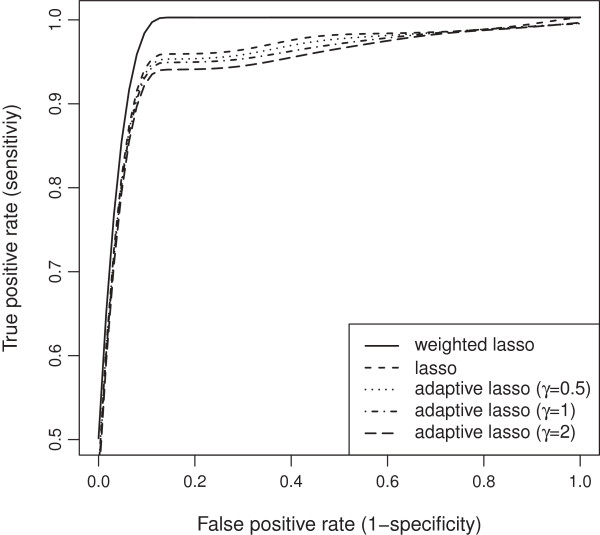
**ROC curves comparison across 5 models for evenly-spaced markers with MCAR mechanism when** ***σ***^**2**^**= 1.**

**Figure 20:**
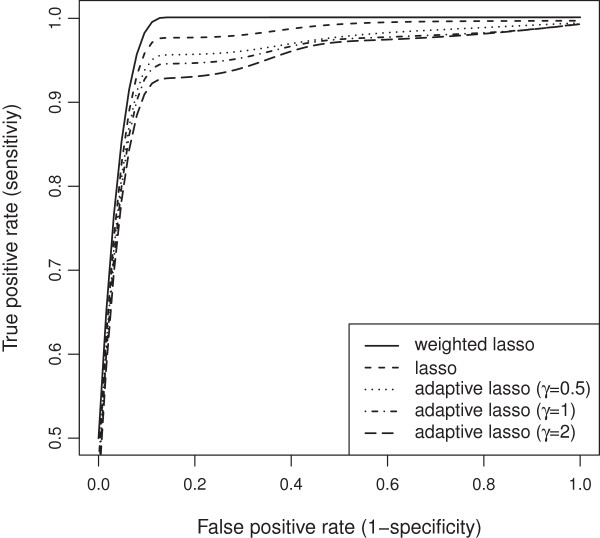
**ROC curves comparison across 5 models for clustered markers with MCAR mechanism when** ***σ***^**2**^**= 1.**

**Figure 21:**
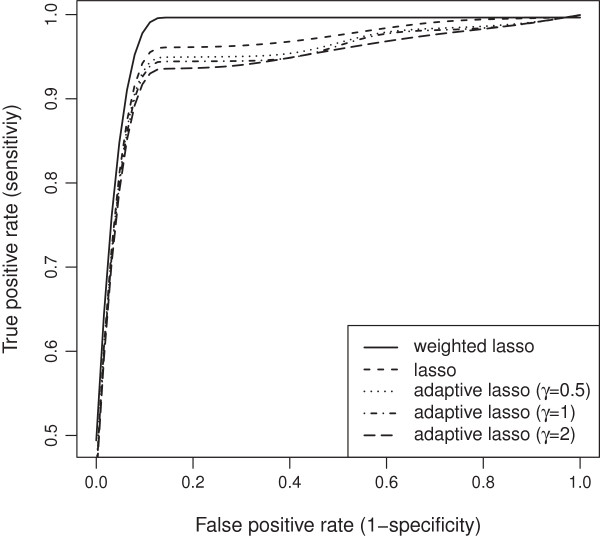
**ROC curves comparison across 5 models for evenly-spaced markers with MAR mechanism when** ***σ***^**2**^**= 1.**

**Figure 22:**
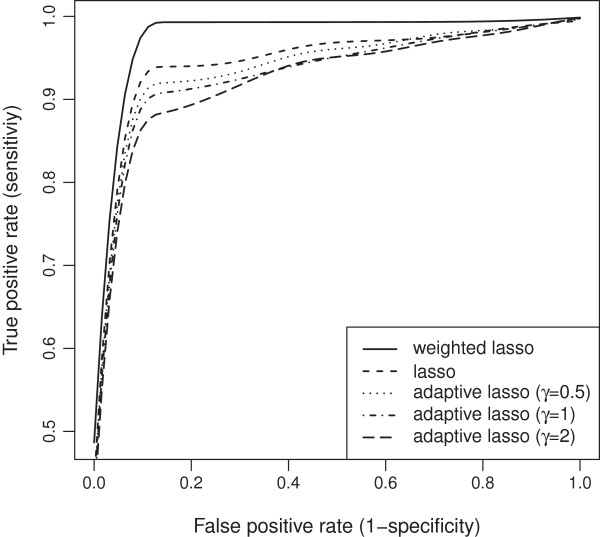
**ROC curves comparison across 5 models for clustered markers with MAR mechanism when** ***σ***^**2**^**= 1.**

Our wlasso is compared with the classical lasso and an adaptive lasso with three weighting schemes [18]. In an adaptive lasso, we estimated the weight vector as ${ŵ}_{\text{adaptive}}=1/|{\hat{\theta }}_{\text{OLS}}{|}^{\gamma }$, where ${\hat{\theta }}_{\text{OLS}}$ is a vector of ordinary least square estimates and *γ*=0.5,1,2 [18]. All five models were applied to the simulated data described above. For lasso and adaptive lasso, the imputed probabilities were rounded towards zeros and ones after the imputation (ignoring our weighting procedure). Thus, an input matrix to lasso and adaptive lasso contained genotype values {0,1}. We investigated settings for equally-spaced and clustered markers with both, MCAR and MAR mechanisms. Again, the results were summarized using ROC curves. The ROC curves of the five models for both, evenly-spaced and clustered markers with MCAR mechanism when the residual error variance *σ*^2^=1 are shown in Figures 19 and 20. Similar plots for MAR are presented in Figures 21 and 22. Clearly, the wlasso is more accurate than other four alternatives. This accuracy is maintained across the investigated variances of all magnitudes (*σ*^2^=0.5,1,2,3), see Additional files 4, 5, 6, 7, 8, 9, 10, 11, 12, 13, 14, and 15. An obvious advantage of wlasso is observed for both, evenly-spaced and clustered markers with MAR mechanism (see Additional files 6, 7, 10, 11, 14, and 15). For clustered markers with MCAR, the wlasso has lost an obvious advantage but still remains at the same accuracy level as lasso when the residual error variance (*σ*^2^=2,3) increases (see Additional files 9 and 13). Though, for evenly-spaced markers with MCAR and large residual error variances (*σ*^2^=2,3), the wlasso maintains noticeably higher accuracy levels than the other four approaches (see Additional files 8 and 12).

## Discussion

The simulation studies have shown that the combination of the proposed probabilistic imputation method and wlasso is an accurate methodology for QTL analysis. The pipeline suggested in this paper has an advantage of computational speed. An alternative to the proposed likelihood-based imputation is multiple imputation [21], but it is slower and leads every time to a possibly different result. The wlasso is used to advance the selection of markers associated with a trait.

In this paper, we analyzed each of the five traits of the Arabidopsis separately. In principle, it is possible to analyse the traits jointly, as they are all traits associated with germination. Clearly, the QTLs shared by all traits can be analyzed further. To identify whether these loci are causal or reactive for a particular trait is an interesting follow-up question. Possible causal relationships among a trio of two traits and a QTL is summarized by others [22]. Their approach can be applied to various pairs of selected Arabidopsis traits and extended to a quintet of traits in order to determine the type of relationship (for example, independent, reactive, causal) existing among traits. Though, this goes beyond the scope of the paper.

## Conclusions

Our methodology has high accuracy in terms of sensitivity and specificity for clustered and evenly-spaced markers for both, MCAR and MAR missing mechanisms. Clearly, the accuracy increases as the magnitude of the residual error variance decreases. In comparison with other approaches, our proposed methodology outperforms alternative methods under most investigated scenarios but is never worse than any of the approaches. More specifically, our probabilistic imputation method is more accurate than the nearest marker imputation. Also, our wlasso is more accurate than commonly practiced multiple regression, the traditional lasso, and adaptive lasso (with the three selected weighting scheme). More importantly, our methodology has been biologically validated on an Arabidopsis study and demonstrated good accuracy. In conclusion, the proposed methodology can be used for QTL identification, especially under the realistic setting of missing genotypes among markers.

## Authors’ contributions

ND conducted the actual work in terms of programming, data analysis and drafting the manuscript. ECW directed the project, assisted in programming and designing the manuscript. ERH provided suggestions on analytical part, reviewed and edited the manuscript. All authors read and approved the final manuscript.

## Supplementary Material

Additional file 1 **Initial and updated weights after 1, 2 and 4 iterations for G**_**max**_**.** Click here for file

Additional file 2 **Initial and updated weights after 1, 2 and 4 iterations for T **_**5** **0**_**.** Click here for file

Additional file 3 **Initial and updated weights after 1, 2 and 4 iterations for T **_**1** **0**_**.** Click here for file

Additional file 4 **ROC curves comparison across 5 models for evenly-spaced markers with MCAR mechanism when** ***σ***^***2***^ ***= 0.5*.** Click here for file

Additional file 5 **ROC curves comparison across 5 models for clustered markers with MCAR mechanism when** ***σ***^***2***^ ***= 0.5*.** Click here for file

Additional file 6 **ROC curves comparison across 5 models for evenly-spaced markers with MAR mechanism when** ***σ***^***2***^ ***= 0.5*.** Click here for file

Additional file 7 **ROC curves comparison across 5 models for clustered markers with MAR mechanism when** ***σ***^***2***^ ***= 0.5*.** Click here for file

Additional file 8 **ROC curves comparison across 5 models for evenly-spaced markers with MCAR mechanism when** ***σ***^***2***^ ***= 2*.** Click here for file

Additional file 9 **ROC curves comparison across 5 models for clustered markers with MCAR mechanism when** ***σ***^***2***^ ***= 2*.** Click here for file

Additional file 10 **ROC curves comparison across 5 models for evenly-spaced markers with MAR mechanism when** ***σ***^***2***^ ***= 2*.** Click here for file

Additional file 11 **ROC curves comparison across 5 models for clustered markers with MAR mechanism when** ***σ***^***2***^ ***= 2***
Click here for file

Additional file 12 **ROC curve comparison across 5 models for evenly-spaced markers with MCAR mechanism when** ***σ***^***2***^ ***= 3*.** Click here for file

Additional file 13 **ROC curves comparison across 5 models for clustered markers with MCAR mechanism when** ***σ***^***2***^ ***= 3***
Click here for file

Additional file 14 **ROC curves comparison across 5 models for evenly-spaced markers with MAR mechanism when** ***σ***^***2***^ ***= 3*.** Click here for file

Additional file 15 **ROC curves comparison across 5 models for clustered markers with MAR mechanism when** ***σ***^***2***^ ***= 3*.** Click here for file
