# Supplementary material for: Probability genotype imputation method and integrated weighted lasso for QTL identification
Source: BMC Genet. 2013 Dec 30;14:125. doi: 10.1186/1471-2156-14-125 (PMC4126192; doi:10.1186/1471-2156-14-125)
Supplement: Additional file 1 — Initial and updated weights after 1, 2 and 4 iterations for G max . [file 1471-2156-14-125-S1.pdf]

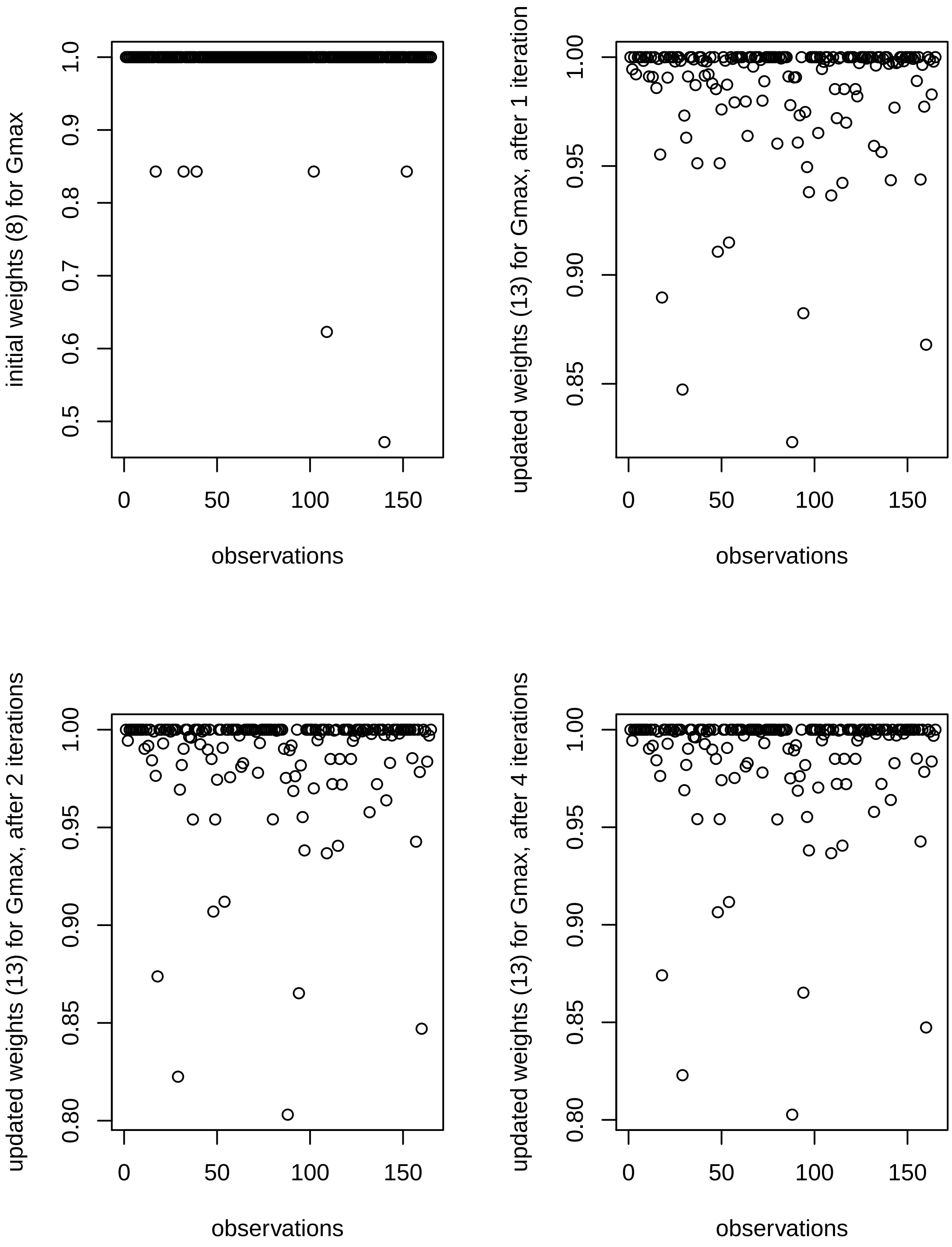

initial weights (8) for Gmax
observations
updated weights (13) for Gmax, after 1 iteration
observations
updated weights (13) for Gmax, after 2 iterations
observations
updated weights (13) for Gmax, after 4 iterations
observations
