# Supplementary material for: Probability genotype imputation method and integrated weighted lasso for QTL identification
Source: BMC Genet. 2013 Dec 30;14:125. doi: 10.1186/1471-2156-14-125 (PMC4126192; doi:10.1186/1471-2156-14-125)
Supplement: Additional file 2 — Initial and updated weights after 1, 2 and 4 iterations for T 5 0 . [file 1471-2156-14-125-S2.pdf]

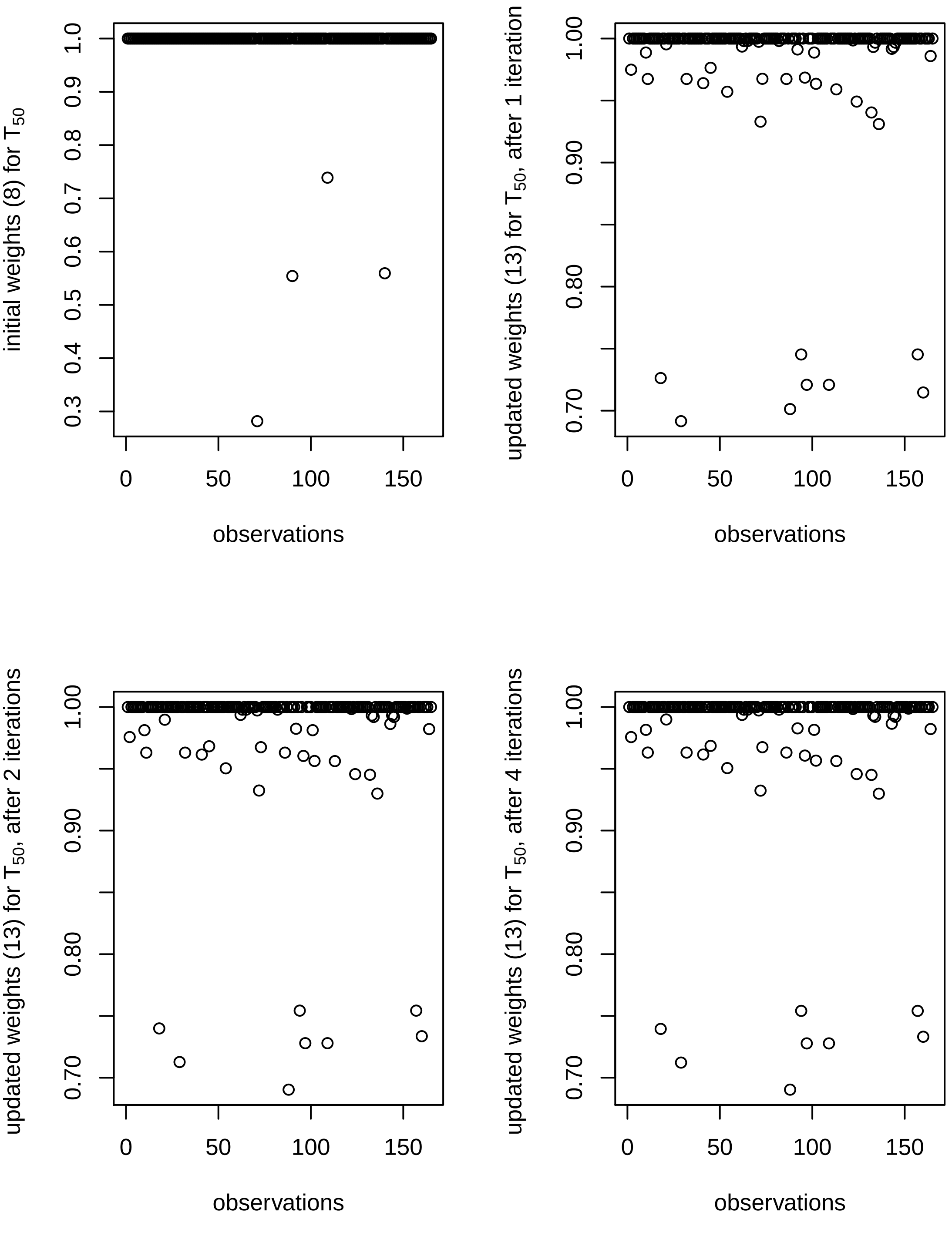
initial weights (8) for T_{50}
1.0
0.9
0.8
0.7
0.6
0.5
0.4
0.3
0
50
100
150
observations
updated weights (13) for T_{50}, after 1 iteration
1.00
0.90
0.80
0.70
0
50
100
150
observations
updated weights (13) for T_{50}, after 2 iterations
1.00
0.90
0.80
0.70
0
50
100
150
observations
updated weights (13) for T_{50}, after 4 iterations
1.00
0.90
0.80
0.70
0
50
100
150
observations
