# Supplementary material for: Probability genotype imputation method and integrated weighted lasso for QTL identification
Source: BMC Genet. 2013 Dec 30;14:125. doi: 10.1186/1471-2156-14-125 (PMC4126192; doi:10.1186/1471-2156-14-125)
Supplement: Additional file 3 — Initial and updated weights after 1, 2 and 4 iterations for T 1 0 . [file 1471-2156-14-125-S3.pdf]

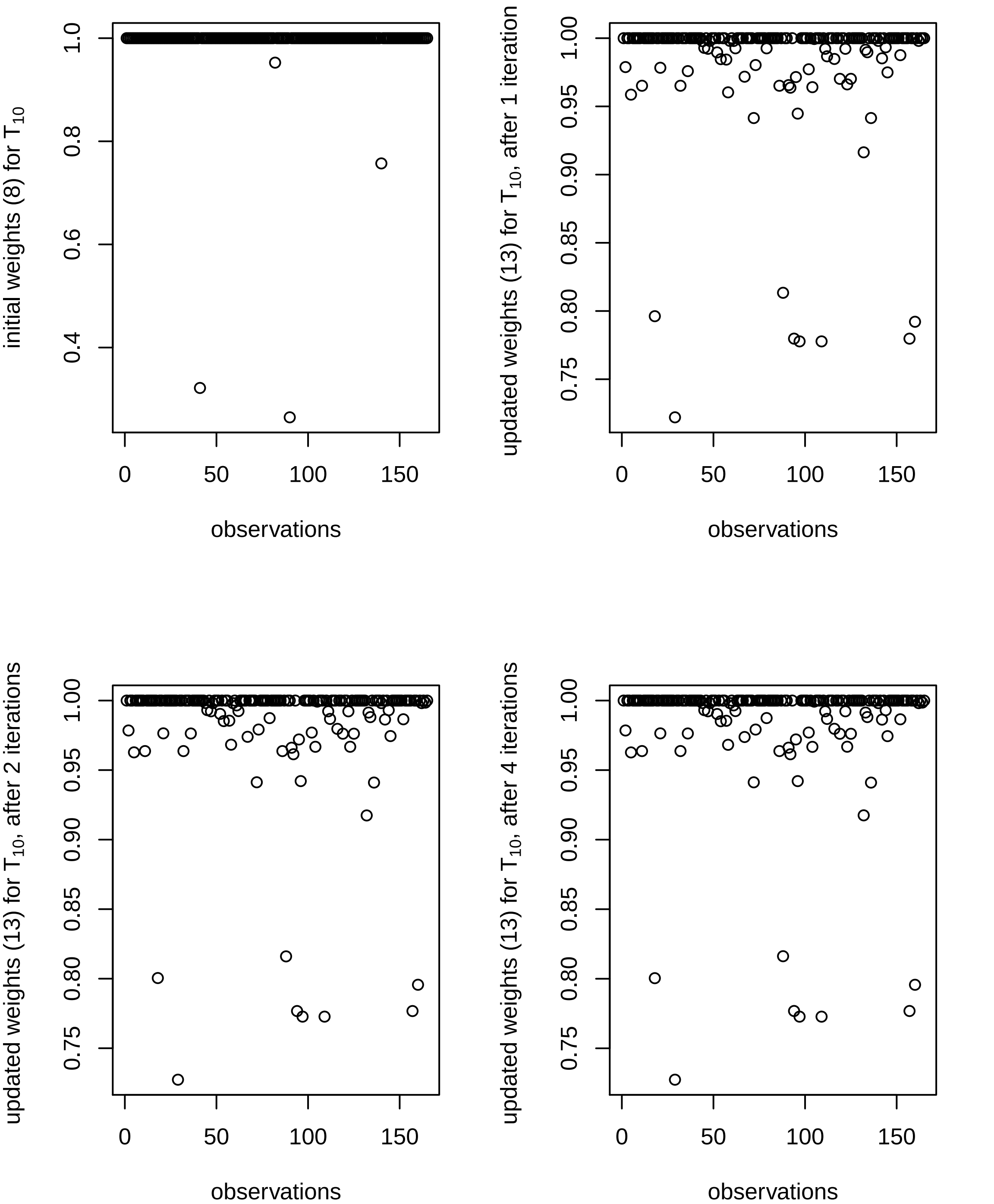
initial weights (8) for T_{10}
updated weights (13) for T_{10}, after 1 iteration
updated weights (13) for T_{10}, after 2 iterations
updated weights (13) for T_{10}, after 4 iterations
observations
