# Supplementary figures and images for: Probability genotype imputation method and integrated weighted lasso for QTL identification
Source: BMC Genet. 2013 Dec 30;14:125. doi: 10.1186/1471-2156-14-125 (PMC4126192; doi:10.1186/1471-2156-14-125)

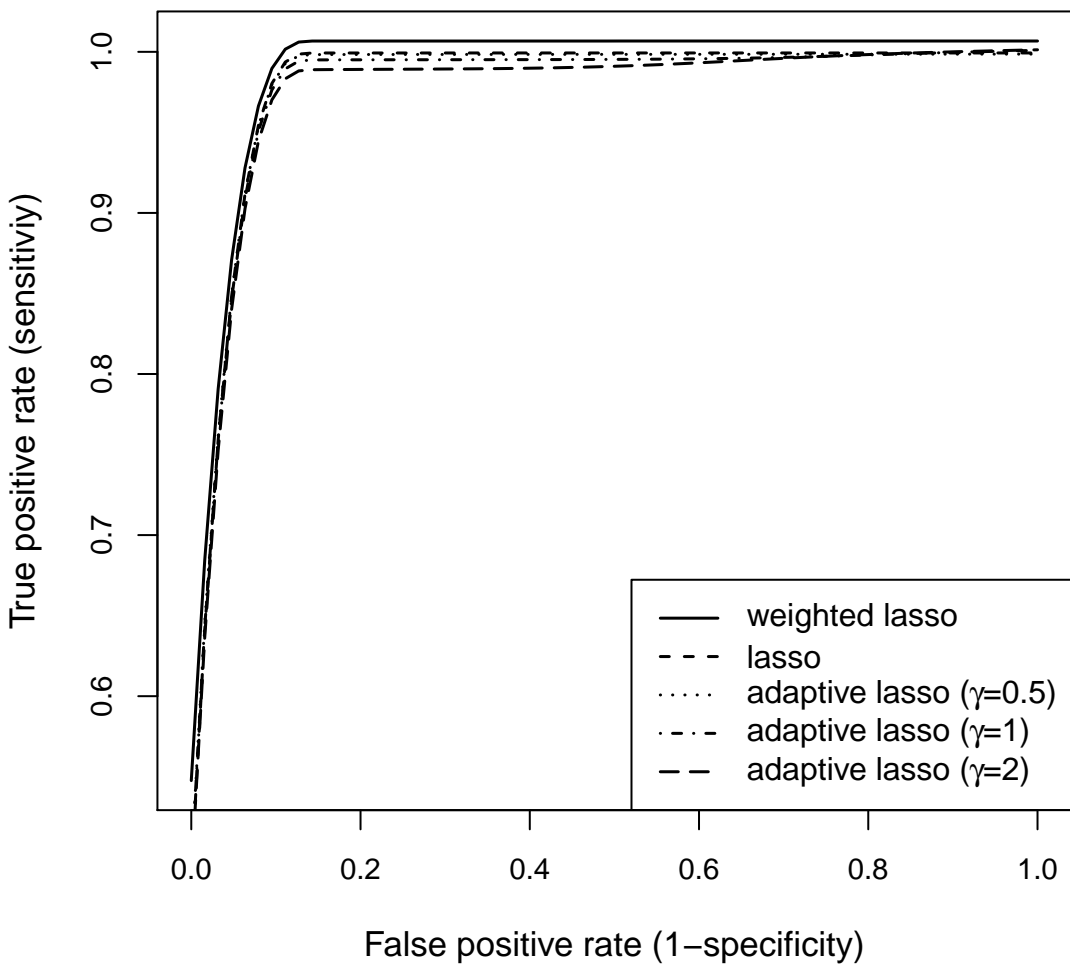

Supplement: Additional file 4 — ROC curves comparison across 5 models for evenly-spaced markers with MCAR mechanism when σ 2 = 0.5 . [file 1471-2156-14-125-S4.pdf]

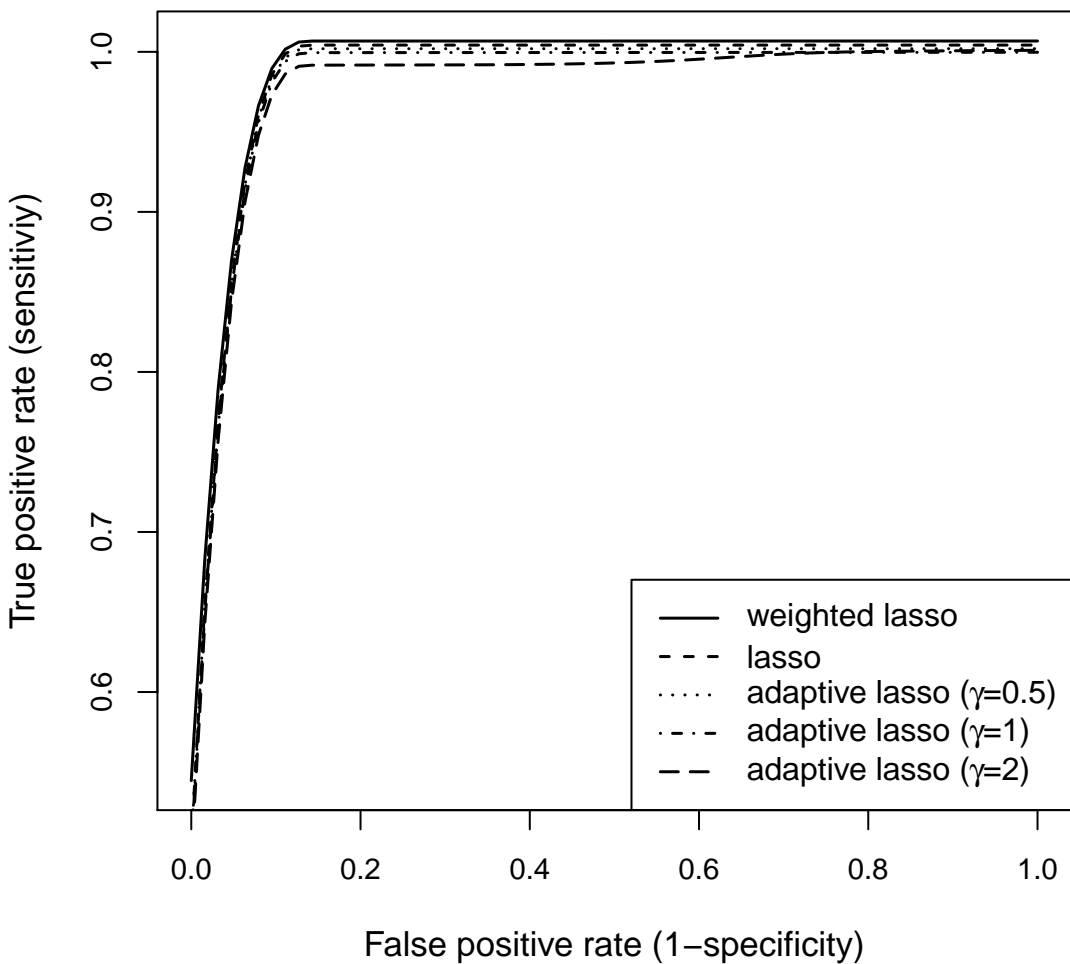

Supplement: Additional file 5 — ROC curves comparison across 5 models for clustered markers with MCAR mechanism when σ 2 = 0.5 . [file 1471-2156-14-125-S5.pdf]

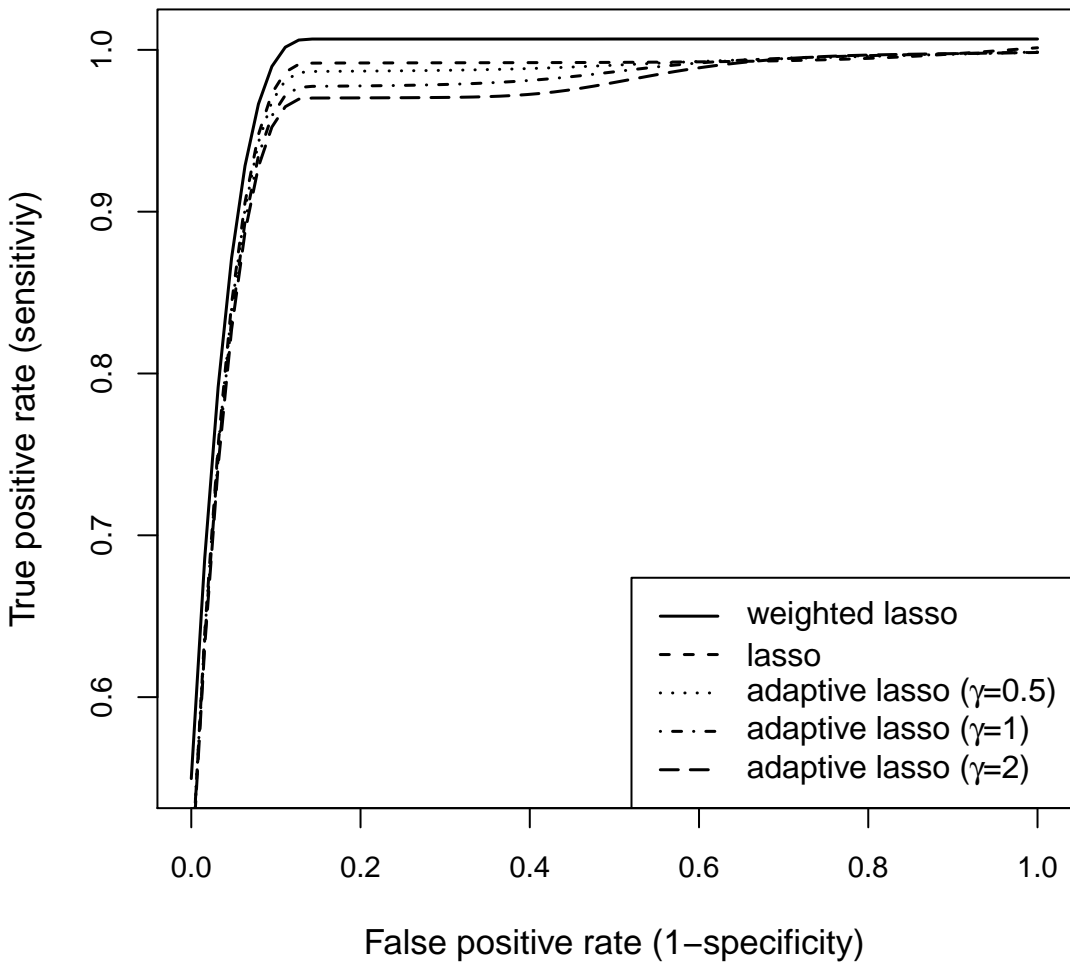

Supplement: Additional file 6 — ROC curves comparison across 5 models for evenly-spaced markers with MAR mechanism when σ 2 = 0.5 . [file 1471-2156-14-125-S6.pdf]

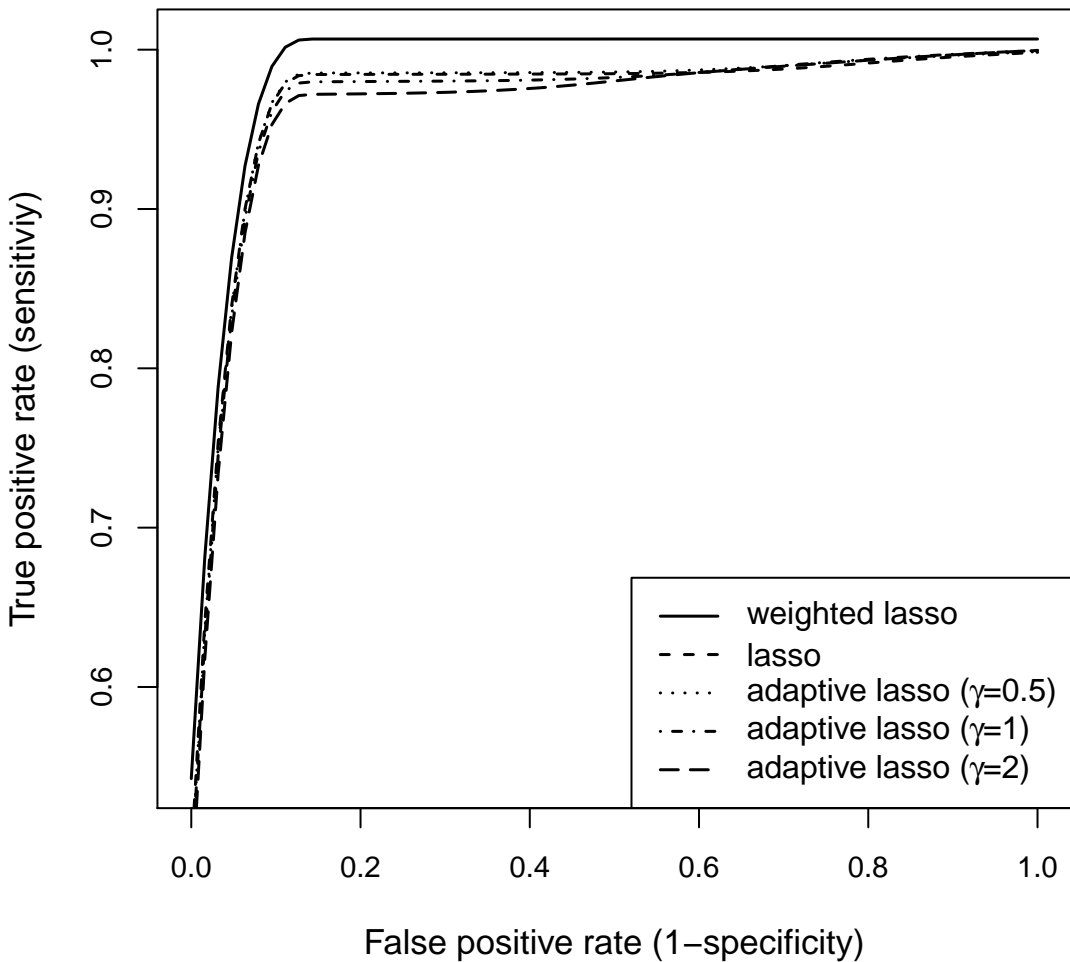

Supplement: Additional file 7 — ROC curves comparison across 5 models for clustered markers with MAR mechanism when σ 2 = 0.5 . [file 1471-2156-14-125-S7.pdf]

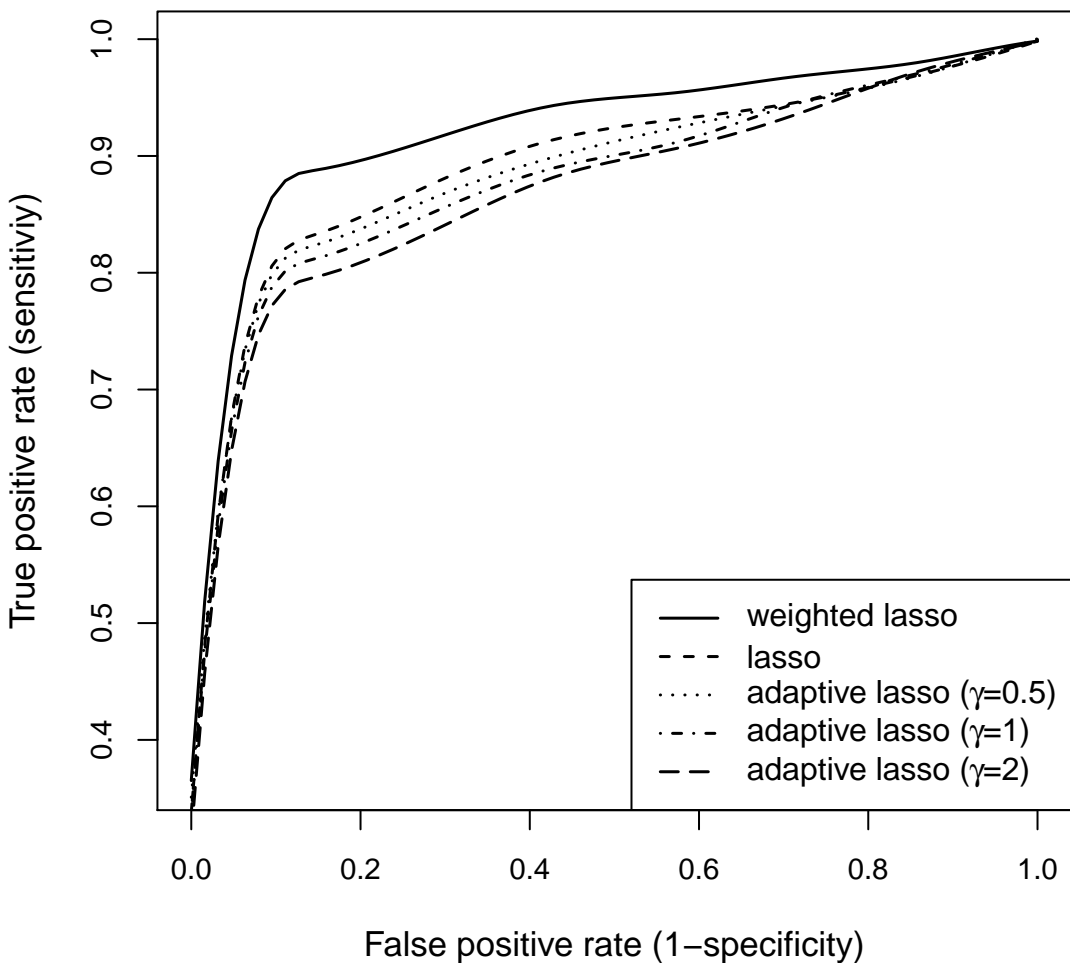

Supplement: Additional file 8 — ROC curves comparison across 5 models for evenly-spaced markers with MCAR mechanism when σ 2 = 2 . [file 1471-2156-14-125-S8.pdf]

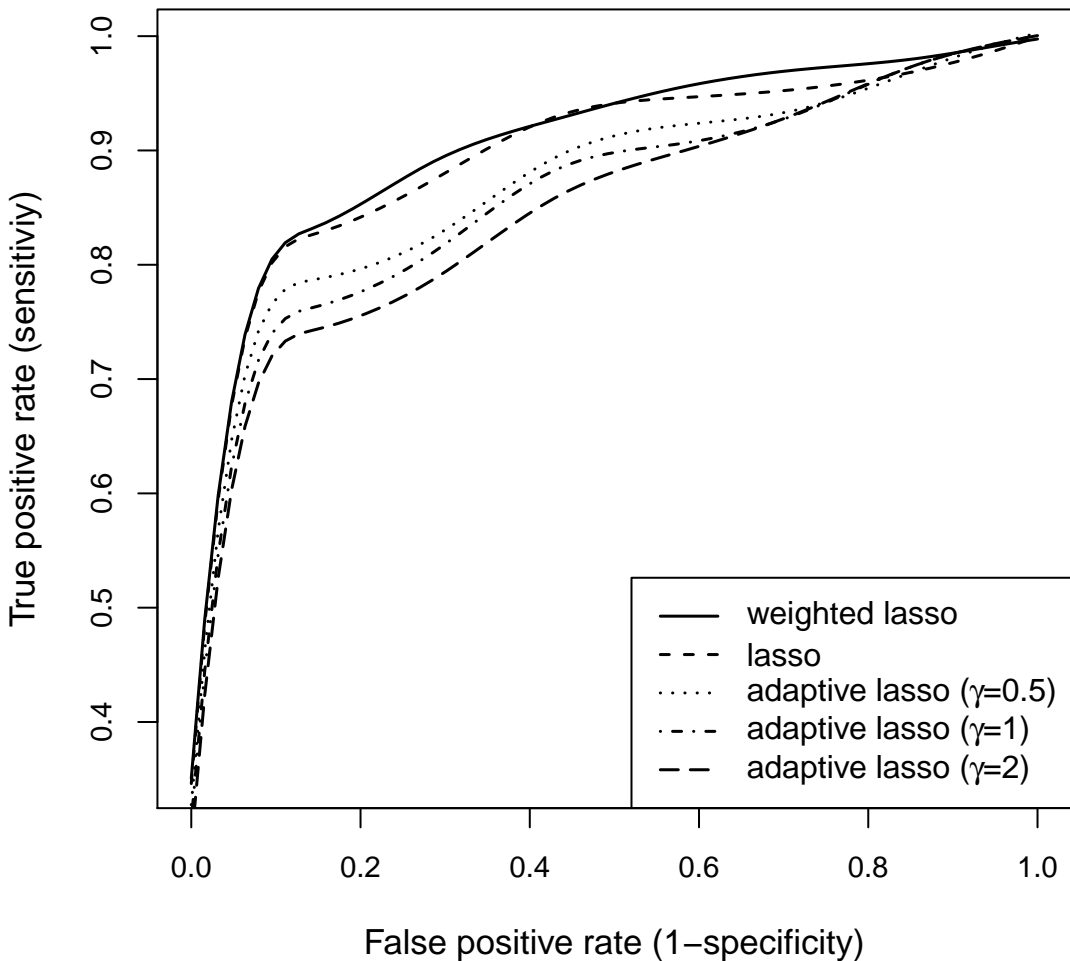

Supplement: Additional file 9 — ROC curves comparison across 5 models for clustered markers with MCAR mechanism when σ 2 = 2 . [file 1471-2156-14-125-S9.pdf]

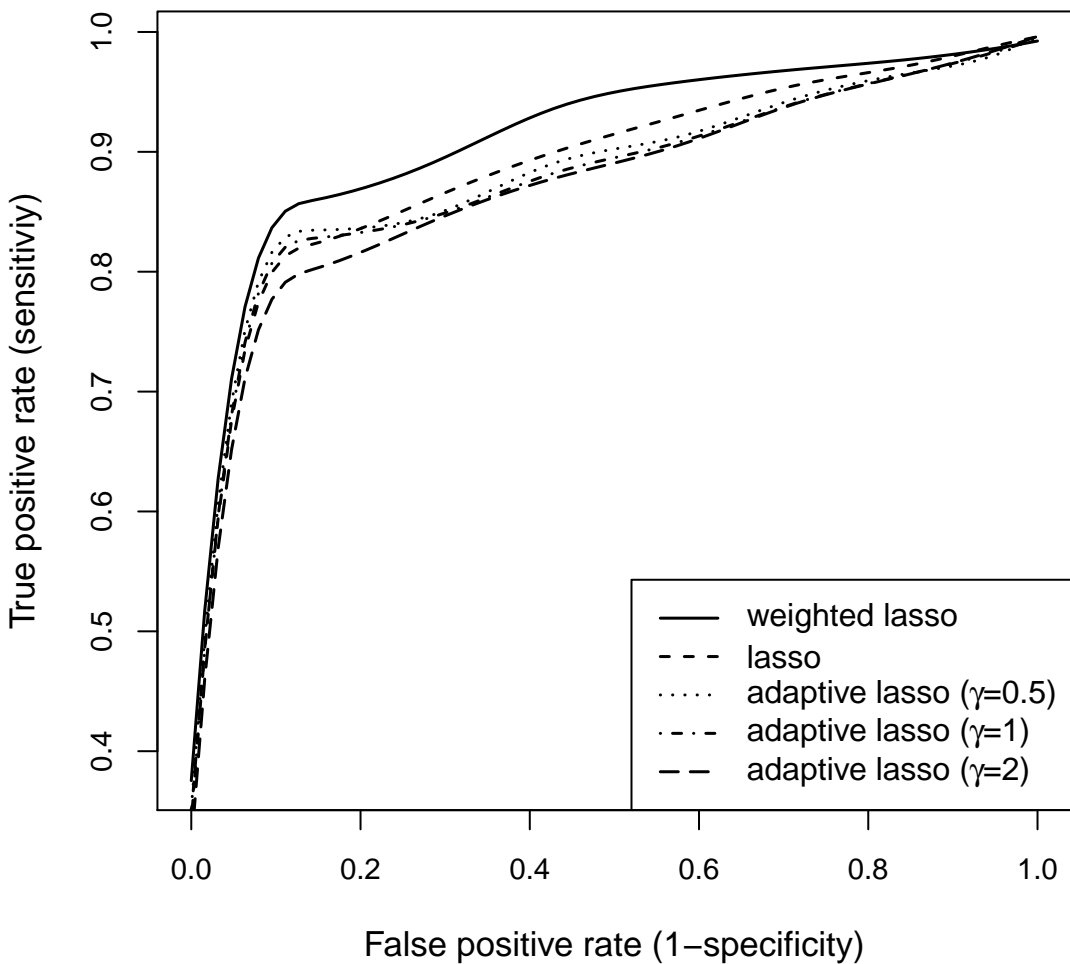

Supplement: Additional file 10 — ROC curves comparison across 5 models for evenly-spaced markers with MAR mechanism when σ 2 = 2 . [file 1471-2156-14-125-S10.pdf]

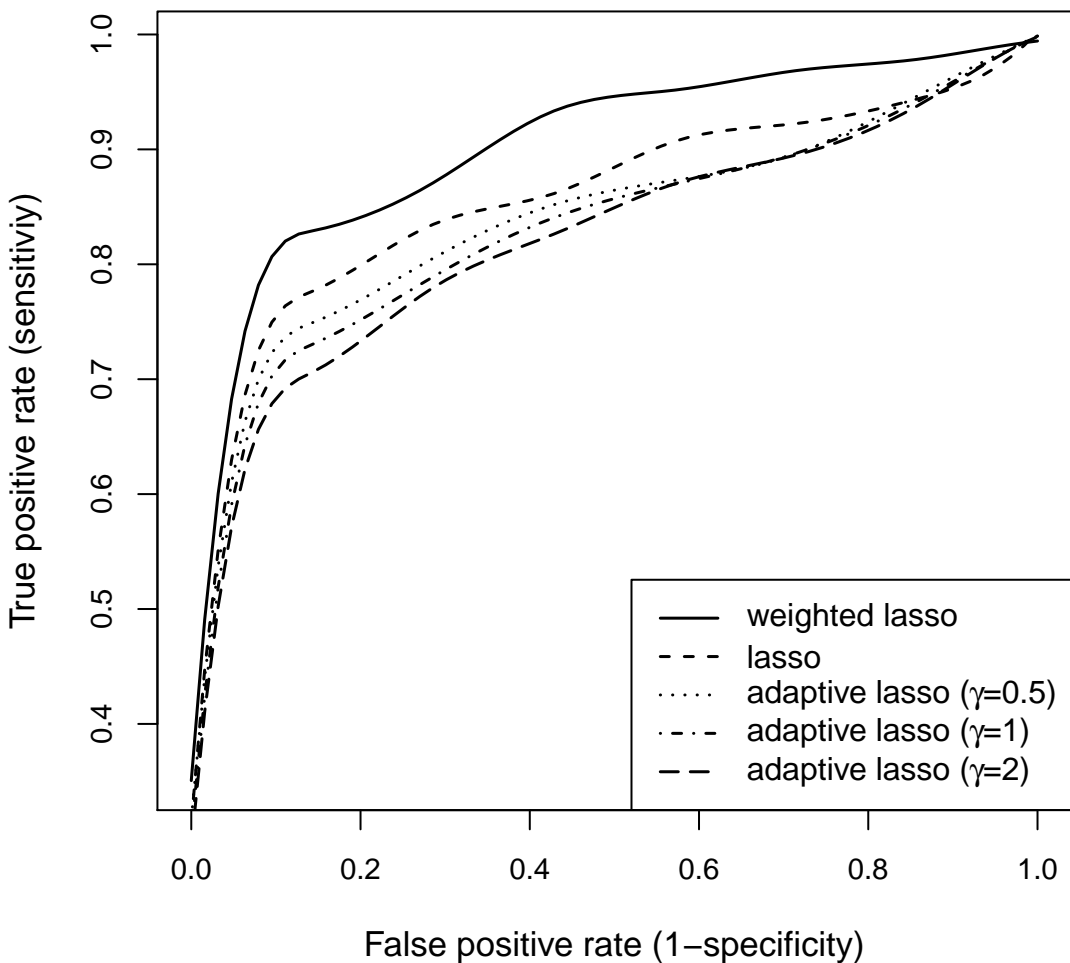

Supplement: Additional file 11 — ROC curves comparison across 5 models for clustered markers with MAR mechanism when σ 2 = 2 [file 1471-2156-14-125-S11.pdf]

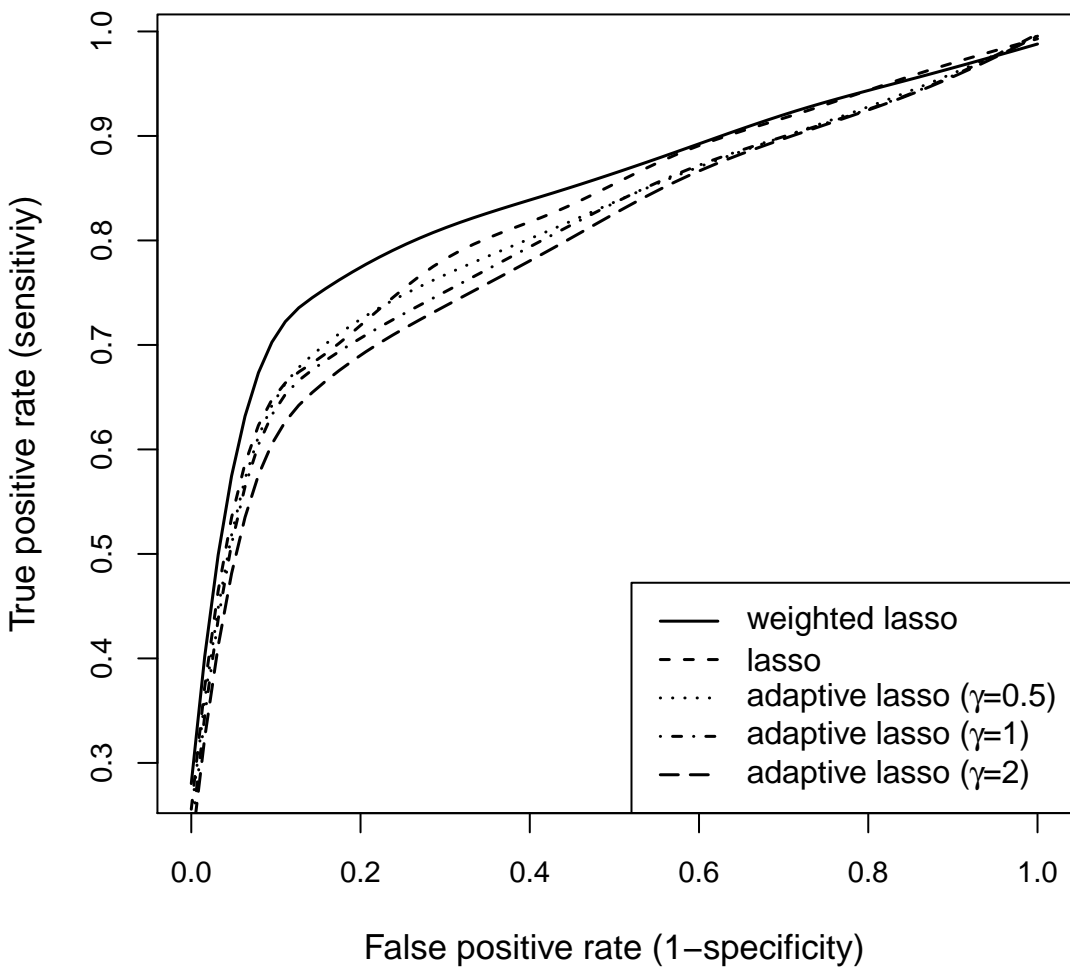

Supplement: Additional file 12 — ROC curve comparison across 5 models for evenly-spaced markers with MCAR mechanism when σ 2 = 3 . [file 1471-2156-14-125-S12.pdf]

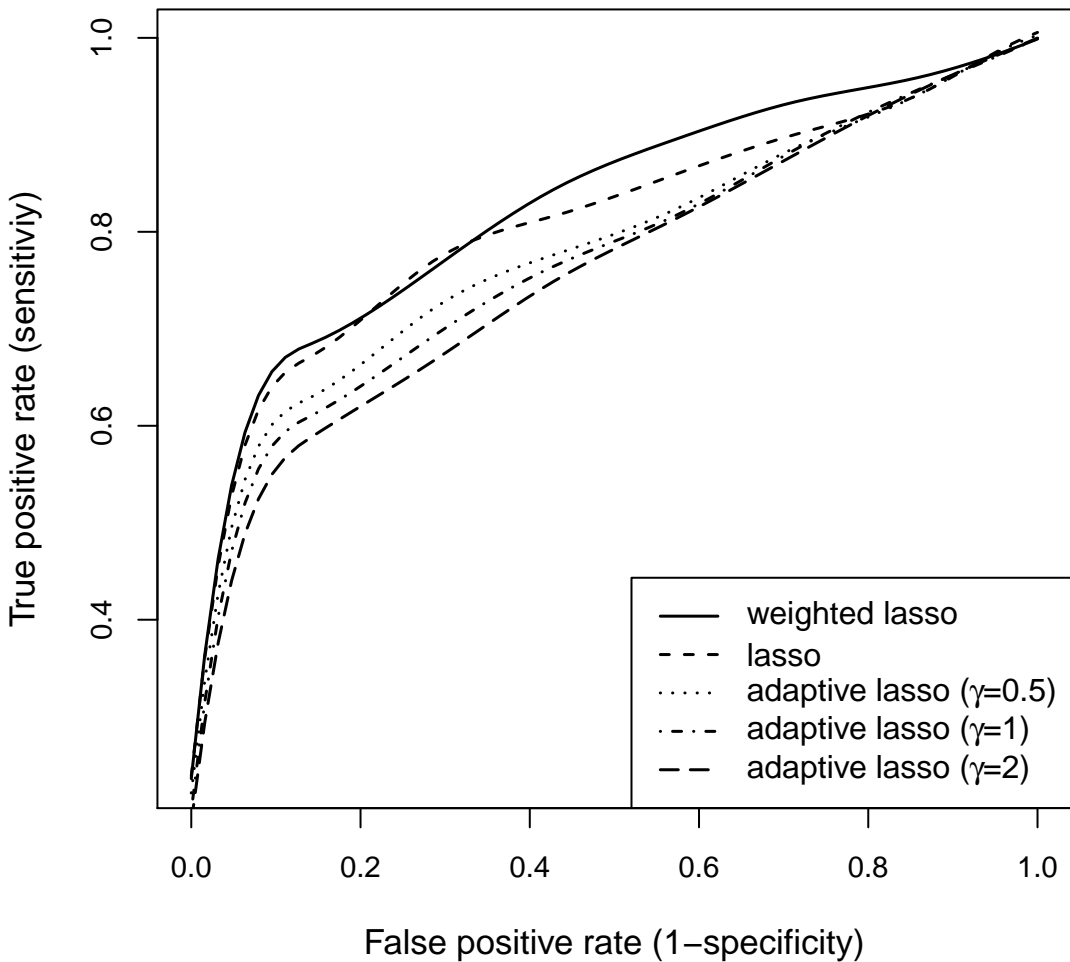

Supplement: Additional file 13 — ROC curves comparison across 5 models for clustered markers with MCAR mechanism when σ 2 = 3 [file 1471-2156-14-125-S13.pdf]

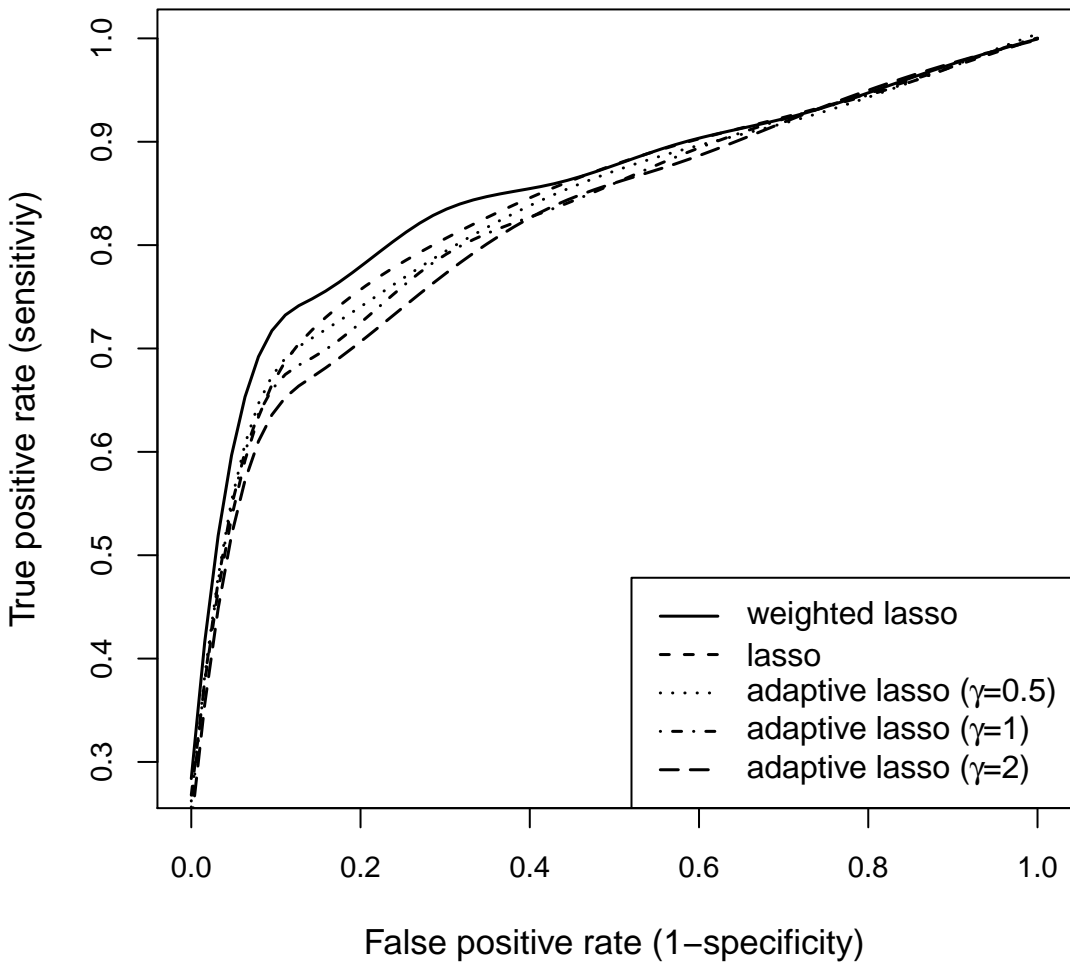

Supplement: Additional file 14 — ROC curves comparison across 5 models for evenly-spaced markers with MAR mechanism when σ 2 = 3 . [file 1471-2156-14-125-S14.pdf]

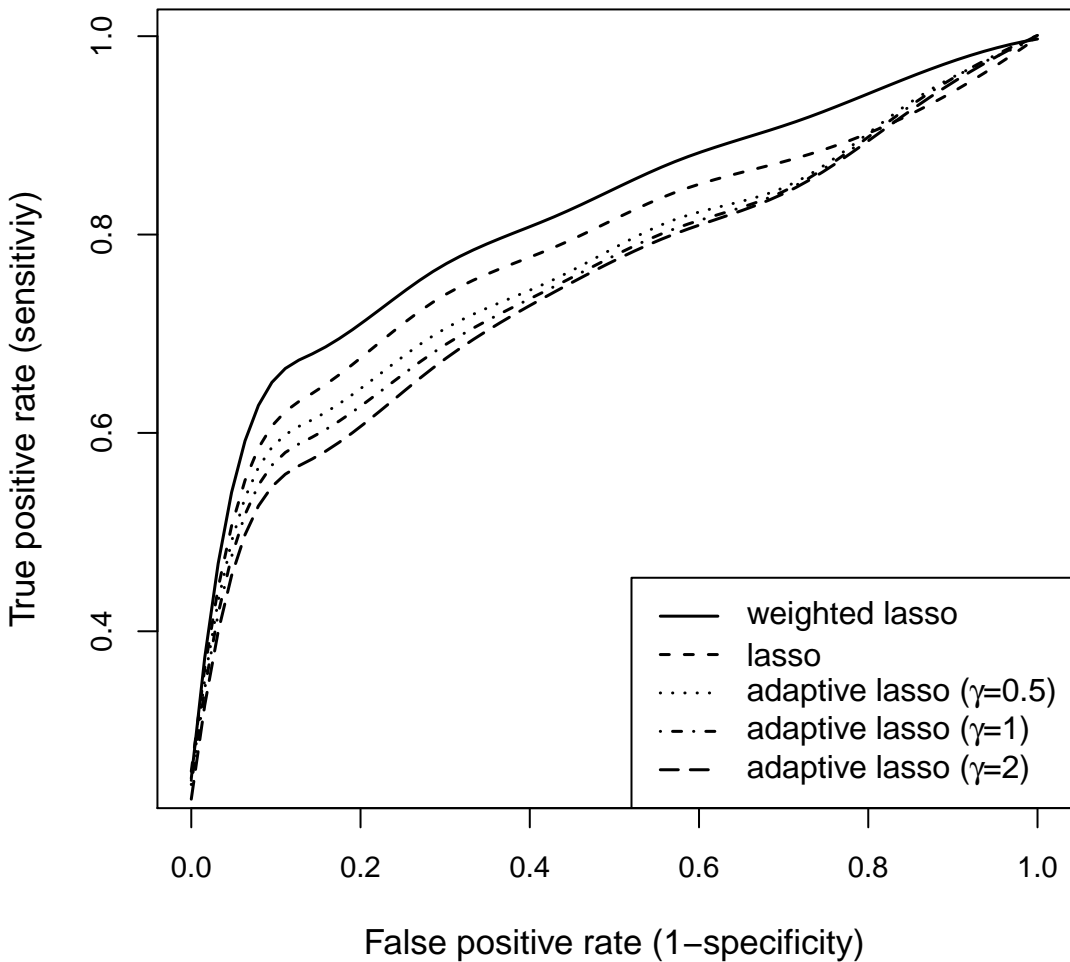

Supplement: Additional file 15 — ROC curves comparison across 5 models for clustered markers with MAR mechanism when σ 2 = 3 . [file 1471-2156-14-125-S15.pdf]
